# Global epidemiology, burden, and causes of lower extremity and pelvic fractures in the past 32 years

**DOI:** 10.3389/fpubh.2025.1627867

**Published:** 2025-07-30

**Authors:** Cun Li, Qingyun Lin

**Affiliations:** ^1^Department of Orthopaedics, The Fifth Affiliated Hospital of Guangxi Medical University, Nanning, China; ^2^Department of Microbiological Testing, Nanning Municipal Center for Disease Control and Prevention, Nanning, China

**Keywords:** lower extremity and pelvic fractures, epidemiology, disease burden, risk factors, GBD 2021

## Abstract

**Background:**

Lower extremity and pelvic fractures (LEPFs) are common and debilitating injuries with substantial global health and economic burden, yet comprehensive epidemiological data remain limited.

**Methods:**

Based on Global Burden of Disease 2021, we analyzed incidence, years lived with disability (YLDs), and causes of LEPFs across 204 countries from 1990 to 2021, along with their temporal trends. The impact of age, sex and Socio-Demographic Index (SDI) was considered.

**Results:**

In 2021, 78.05 million new LEPF cases were reported globally, a 32% increase since 1990. Despite this, the age-standardized incidence rate (ASIR) declined annually by 0.68%, reaching 974.98 per 100,000 population in 2021. For anatomic subtypes, fractures of the patella, tibia or fibula, or ankle were most common (34.96 million), while hip fractures showed the largest increase for incident cases (126%) and a significant rising ASIR among males (0.21% annually). Conflict-affected countries in the Middle East and Africa saw the sharpest rises in LEPF burden. SDI correlated with elevated ASIR and age-standardized YLD rates (ASYR), particularly at SDI > 0.7. Falls were the leading cause, followed by road injuries. YLDs peaked among adults aged 45–60, with ASYR rising sharply in older populations. The disability burden increased during COVID-19 pandemic, exposing vulnerabilities in global fracture care systems.

**Conclusion:**

LEPFs remain a significant public health challenge, driven by population aging, regional instability and osteoporosis. Hip fractures in males and LEPFs in conflict zones demand urgent attention. Strengthening fall prevention, implementing comprehensive osteoporosis management including sex-inclusive approaches, and targeted prevention strategies is essential to mitigating the global burden of LEPFs.

## Introduction

Lower extremity and pelvic fractures (LEPFs) account for approximately 30% of all fractures, significantly impairing patients’ mobility and joint function, and may lead to severe complications such as shock, deep vein thrombosis, systemic organ damage, and infections, thereby posing substantial global health and economic burdens (1–3). A study based on the Danish National Patient Registry reported 30-day mortality rates of 10% for femoral fractures, 2% for tibial fractures, and 1% for foot and ankle fractures (4). Regarding functional outcomes, LEPFs profoundly impact on patients’ functional autonomy. Clinical investigations reveal that more than 60% of femur fracture patients experience reduced self-sufficiency, requiring external support for routine tasks. Longitudinal follow-up data indicate persistent care dependency among these patients, with 33% still requiring care-giving support one year after the injury (5, 6). From an economic perspective, a study from Netherlands reported that LEPFs accounted for the highest proportion of injury-related medical expenses, with hip fractures alone costing an average of €20,000 per patient (7). Another study based on Medical Expenditure Panel Survey in the U. S. found that the total expenditure and out-of-pocket costs associated with LEPFs have increased significantly over the past decade, outpacing the growth of general health-care costs (8).

The risk factors contributing to LEPFs are multifactorial, encompassing age, sex, deficiencies in calcium and vitamin D, osteoporosis, as well as traumatic events (3). Among these, trauma stands out as the primary cause of LEPFs, and implementing targeted strategies to mitigate trauma-related risks appears to be a practical and achievable approach (9). Furthermore, osteoporosis, characterized by reduced bone density and microarchitectural deterioration, is a common condition in the older adult population and significantly increases skeletal fragility, thereby elevating the risk of LEPFs even with low-impact trauma (10). In addition, prior research has demonstrated a correlation between the incidence of hip fractures and socioeconomic development (6). However, the association between socioeconomic status and other subtypes of LEPFs remains unclear. In view of these factors, there is a pressing need for a comprehensive and up-to-date epidemiological assessment of LEPFs. Nevertheless, existing studies have been predominantly limited to single-country analyses, and many countries worldwide still lack related data on LEPFs (11). Moreover, no studies have assessed the disability burden associated with LEPFs at different anatomical sites, and current research lacked comprehensive evaluations of relevant risk factors.

The Global Burden of Disease Study 2021 (GBD 2021), representing the most up-to-date and comprehensive global resource on disease epidemiology and burden, encompasses data on 459 health outcomes and 88 risk factors across 204 countries and territories from 1990 to 2021 (12). In this study, we utilized the updated GBD 2021 data to conduct a comprehensive epidemiological assessment of LEPFs, analyze age- and sex-specific disparities, examine inequalities in disease burden and associated risk factors, with the aim of informing evidence-based and targeted policy-making.

## Methods

### Data and measures

In this analysis, based on the GBD 2021 obtained from the Global Health Data Exchange (https://vizhub.healthdata.org/gbd-results/), we focused on LEPFs, including the total LEPFs and the following anatomic sites: (1) pelvic; (2) hip; (3) femur, excluding femoral neck; (4) patella, tibia or fibula, or ankle; and (5) foot bones excluding the ankle. We specifically extracted data on the incidence and years lived with disability (YLD) of LEPFs, including the number of cases and age-standardized rates, to assess the epidemiology and burden of LEPFs. The number of incident cases represents the total count of newly diagnosed cases within a given year, whereas the age-standardized incidence rate (ASIR) reflects the proportion of new cases relative to the population at risk. YLDs represent the number of years lived with disability attributable to LEPFs, capturing the duration and severity of health loss, and thus indicating their impact on quality of life. The age-standardized YLD rate (ASYR) indicates the proportion of YLDs in the at-risk population. These measures were presented as point estimates with their corresponding 95% uncertainty intervals (UIs) (13).

### Percentage change and estimated annual percentage change

To illustrate the total trends in the total number of cases, we calculated the percentage change (PC) in incident cases and YLDs for LEPFs from 1990 to 2021, using the following formula:


$$PC_{\left(1990-2021\right)}=(\text{Case}s_{2021}-\text{Case}s_{1990})/\text{Number of Case}s_{1990}$$

To assess the average annual trend in the ASIR and ASYR of LEPFs, we used the Estimated Annual Percentage Change (EAPC). The EAPC is calculated by fitting a linear regression model to the natural logarithm of the rates (e.g., ASIR or ASYR), with the calendar year as the independent variable. The formula is as follows:


$$\ln\;(\text{Rates})=\alpha +\beta^{*}\text{Year}+\sigma .$$

EAPC=(exp(β)−1)∗100.

In this equation, the sign of the EAPC corresponds to the sign of the regression coefficient (β), indicating the direction of the trend in the measured indicator over time. The 95% confidence interval (CI) of the EAPC is derived from the standard error of the regression coefficient.

### Causes of injury

In the GBD 2021 framework, the causes of diseases and injuries are categorized hierarchically into four levels. Level 1 causes represent broad categories, including Non-communicable diseases, Communicable diseases, Maternal, neonatal, and nutritional diseases, and Injuries. Causes of fractures are primarily categorized under the Injuries section. Our analysis focuses on Level 3 causes under the Injuries category, which includes 16 distinct causes such as falls, road injuries, exposure to mechanical forces, interpersonal violence, self-harm, and natural disasters, among others. We concentrated on the top five causes that contribute most significantly to LEPFs and their anatomic subtypes.

### Correlation analysis between socio-demographic index and LEPFs

The Socio-demographic Index (SDI) is a composite measure used to reflect the level of social and demographic development of a given region. The calculation of the SDI is based on three key indicators: the total fertility rate among women under the age of 25, the mean years of education among individuals aged 15 years and older, and per capita income. Each region is assigned an SDI score ranging from 0 to 1 (13).

We employed both linear regression and Locally Estimated Scatterplot Smoothing (LOESS) to examine the relationship between SDI and the ASIR or ASYR of LEPFs. LOESS is a non-parametric regression method that fits the data locally to capture potential non-linear trends (14). The combination of linear regression and LOESS allows for a more accurate identification of complex relationships across the SDI spectrum. A LOESS curve was fitted to visualize the overall trend (black line). The results of linear regression are also shown (regression coefficient (*β*), coefficient of determination (*R*^2^), and *p*-value), indicating an overall association. β, *R*^2^, and p-value were used to assess the explanatory power, correlation, and significance, respectively. A p-value less than 0.004 (0.05/12) was considered statistically significant.

## Results

### Global epidemiology and burden of LEPFs

**Figure 1:**
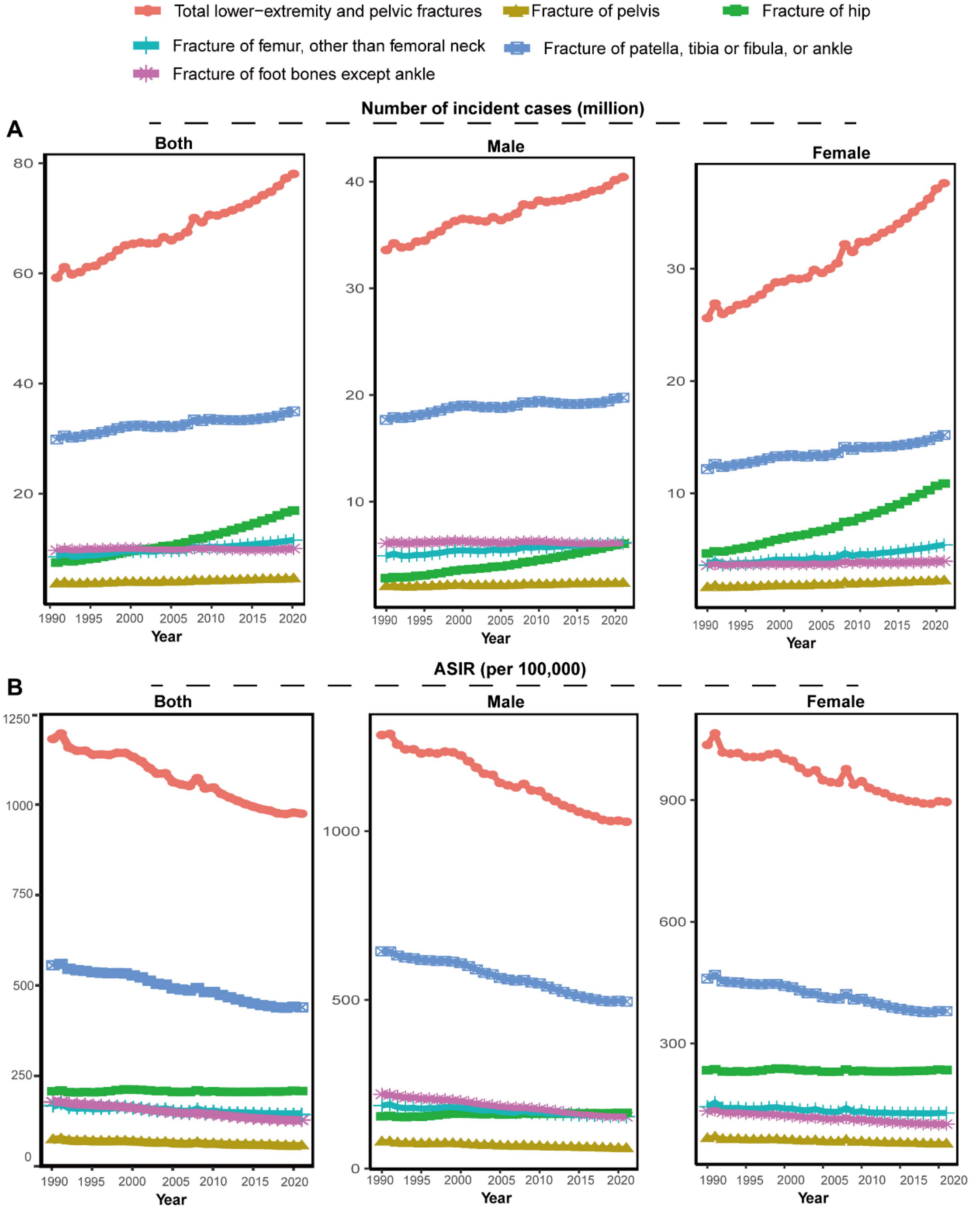
The number of incident cases and ASIR for total LEPFs and anatomical subtypes by sex, 1990–2021. **(A)** The number of incident cases. **(B)** ASIR per 100,000 population. ASIR, age-standardized incidence rate; LEPFs, lower extremity and pelvic fractures.

Globally, from 1990 to 2021, the number of incident cases of total LEPFs and all anatomical subtypes increased (Figure 1A; Table 1). Incident cases of total LEPFs rose by 32% overall, with a 20% increase in males and a 47% increase in females. Notably, hip fractures showed the largest relative increase—126% overall, 114% in females, and 134% in males. In 2021, there were 78.05 million LEPFs globally, with fractures of the patella, tibia or fibula, or ankle accounting for the highest number (34.96 million cases) (Table 1).

**Table 1:** Global Incident cases and ASIR of LEPFs between 1990 and 2021, and their temporal trends.

Fracture site	Number of incident cases			ASIR		
	1990 (95% UI)	2021 (95% UI)	PC	1990 (95% UI)	2021 (95% UI)	EAPC (95% CI)
Total LEPFs						
Both	59,202,582 (47,687,840, 73,526,077)	78,046,598 (61,229,471, 99,113,574)	0.32	1181.58 (944.65, 1473.31)	974.98 (766.6, 1235.14)	−0.68 (−0.72, −0.64)
Female	25,608,835 (19,802,920, 32,953,747)	37,615,184 (28,190,952, 49,124,083)	0.47	1036.16 (797.89, 1334.55)	895.37 (673.38, 1168.39)	−0.54 (−0.59, −0.5)
Male	33,593,748 (27,590,847, 40,964,709)	40,431,413 (32,881,434, 50,063,036)	0.2	1285.09 (1054.22, 1569.57)	1028.04 (835.46, 1272.49)	−0.77 (−0.81, −0.73)
Fracture of pelvis						
Both	3,604,239 (2,794,736, 4,801,902)	4,524,448 (3,283,345, 6,583,735)	0.26	73.04 (55.6, 98.92)	56 (40.96, 81.22)	−0.86 (−0.92, −0.81)
Female	1,581,120 (1,155,532, 2,186,808)	2,188,606 (1,462,434, 3,344,409)	0.38	64.91 (46.34, 92)	51.55 (34.73, 77.11)	−0.78 (−0.86, −0.71)
Male	2,023,119 (1,618,229, 2,599,340)	2,335,842 (1,828,366, 3,216,754)	0.15	78.53 (62.44, 102.37)	58.99 (46.01, 81.58)	−0.9 (−0.95, −0.85)
Fracture of hip						
Both	7,483,727 (5,789,986, 9,731,435)	16,941,491 (12,748,027, 22,170,685)	1.26	207.54 (157.98, 268.09)	208.27 (157.02, 271.93)	−0.01 (−0.05, 0.03)
Female	4,645,722 (3,475,067, 6,151,968)	10,870,656 (8,004,569, 14,177,299)	1.34	234 (174.71, 307.98)	234.62 (173.11, 306.92)	−0.01 (−0.05, 0.03)
Male	2,838,005 (2,233,747, 3,620,060)	6,070,835 (4,698,684, 7,939,123)	1.14	154.98 (121.29, 198.81)	165.58 (127.99, 215.87)	0.21 (0.16, 0.26)
Fracture of femur, other than femoral neck						
Both	8,559,886 (7,018,864, 10,645,221)	11,566,429 (8,890,197, 15,048,861)	0.35	167.75 (136.18, 210.54)	143.2 (110.54, 185.62)	−0.57 (−0.63, −0.51)
Female	3,623,680 (2,867,566, 4,656,549)	5,408,470 (3,939,064, 7,168,481)	0.49	144.12 (112.07, 185.54)	128.5 (94.24, 170.02)	−0.46 (−0.54, −0.38)
Male	4,936,206 (4,078,136, 6,089,809)	6,157,959 (4,982,323, 7,777,555)	0.25	187.13 (154.16, 230.99)	154.42 (125.23, 194.59)	−0.64 (−0.69, −0.59)
Fracture of patella, tibia or fibula, or ankle						
Both	29,834,211 (24,615,732, 35,568,313)	34,958,294 (28,619,051, 42,091,476)	0.17	555.63 (458.81, 662.39)	439.66 (360.02, 528.34)	−0.84 (−0.89, −0.8)
Female	12,162,025 (9,616,117, 15,098,765)	15,195,315 (11,864,039, 19,010,424)	0.25	459.76 (365.22, 568.27)	379.63 (296.5, 475.54)	−0.72 (−0.76, −0.67)
Male	17,672,186 (14,874,232, 20,814,881)	19,762,979 (16,616,132, 23,361,845)	0.12	644.15 (543.84, 754.79)	495.26 (416.58, 585.28)	−0.93 (−0.97, −0.89)
Fracture of foot bones except ankle						
Both	9,720,519 (7,468,522, 12,779,206)	10,055,936 (7,688,851, 13,218,817)	0.03	177.62 (136.08, 233.37)	127.85 (98.06, 168.03)	−1.12 (−1.15, −1.08)
Female	3,596,288 (2,688,638, 4,859,657)	3,952,137 (2,920,846, 5,423,470)	0.1	133.37 (99.55, 180.76)	101.07 (74.8, 138.8)	−0.96 (−1, −0.92)
Male	6,124,232 (4,786,503, 7,840,619)	6,103,798 (4,755,929, 7,767,759)	0	220.3 (172.49, 282.61)	153.79 (119.65, 195.17)	−1.21 (−1.24, −1.18)

ASIR, age-standardized incidence rate; EAPC, estimated annual percentage change; LEPFs, lower extremity and pelvic fractures; PC, percentage change; UI, uncertainty intervals; CI, confidence interval.

From 1990 to 2021, the ASIR of total LEPFs and nearly all anatomical subtypes—except for hip fractures—declined in both sexes (Figure 1B). The ASIR of total LEPFs declined at an average annual rate of 0.68% (EAPC: −0.68, 95% CI: −0.72 to −0.64), with a more pronounced decline in males (EAPC: −0.77, 95% CI: −0.81 to −0.73). Among anatomical subtypes, the greatest ASIR decline was observed in foot fractures, decreasing by 1.12% annually overall (1.21% in males, 0.96% in females). In contrast, the ASIR of hip fractures remained stable in females and the total population (EAPC: −0.01, 95% CI: −0.05 to 0.03), but showed an annual increase of 0.21% in males (EAPC: 0.21, 95% UI: 0.16 to 0.26), making it the only subtype with an upward trend. In 2021, the ASIR of total LEPFs was 974.98 per 100,000 population—1028.04 in males and 895.37 in females. Among anatomical subtypes, the highest ASIR was seen in fractures of the patella, tibia or fibula, or ankle (439.66 per 100,000), followed by hip (208.27 per 100,000), femur (143.2 per 100,000), foot (127.85 per 100,000), and pelvis (56 per 100,000). ASIRs were higher in males than in females across all subtypes except hip fractures (Table 1).

Globally, from 1990 to 2021, the number of YLDs of total LEPFs and all subtypes increased, mirroring trends in incidence (Supplementary Figure S1A). However, the ASYR of total LEPFs and all subtypes decreased. The largest decline in ASYR was observed in pelvic fractures, with an average annual reduction of 1.3% overall (EAPC: −1.3, 95% CI: −1.37 to −1.23), 1.34% for male (EAPC:-1.34, 95% CI: −1.42 to −1.27), and 1.24% for female (EAPC:-1.24, 95% CI: −1.31 to −1.27) (Supplementary Figure S1B; Supplementary Table S1). Notably, the ASYR of total LEPFs showed an upward trend after 2019 (Supplementary Figure S1B).

### Epidemiology and burden of LEPFs across 204 countries and territories

In 2021, in terms of the incident cases, femoral fractures had the highest counts in China, India, and Russia. For total LEPFs and other anatomic types, the highest incident cases were observed in China, India, and the United States (Supplementary Figure S2). Regarding the percentage change in incident cases between 1990 and 2021, the largest increases were found in Middle Eastern and African countries, such as the United Arab Emirates, Afghanistan, Qatar, and Yemen (Supplementary Figure S3). Notably, for hip fractures, substantial percentage increases were also observed in Canada, China, and countries in Oceania (Supplementary Figure S3C).

**Figure 2:**
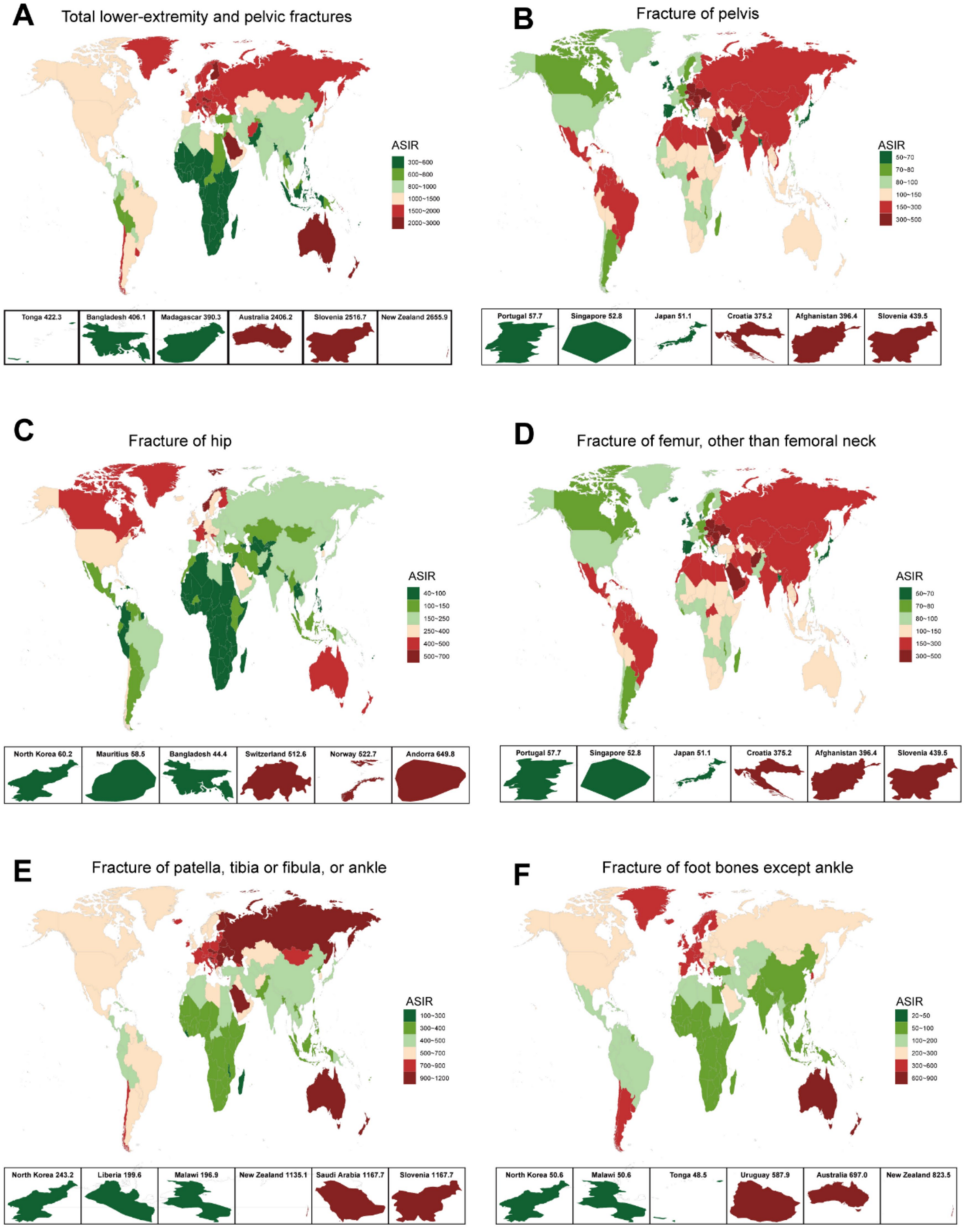
ASIR for total LEPFs and anatomical subtypes across 204 countries and territories in 2021. **(A)** The overall LEPFs. **(B)** Fracture of pelvis. **(C)** Fracture of hip. **(D)** Fracture of femur, other than femoral neck. **(E)** Fracture of patella, tibia or fibula, or ankle. **(F)** Fracture of foot bones except ankle. ASIR, age-standardized incidence rate; LEPFs, lower extremity and pelvic fractures.

In 2021, the ASIR of LEPFs varied across countries, with the highest rates primarily observed in developed economies as classified by the World Bank. For total LEPFs, the top three countries with the highest incidence rates were New Zealand (2,655.9 per 100,000), Slovenia (2,516.7 per 100,000), and Australia (2,406.2 per 100,000). For pelvic fractures, the countries with the highest incidence rates were Slovenia (439.5 per 100,000), Afghanistan (396.4 per 100,000), and Croatia (375.2 per 100,000). For hip fractures, the countries with the highest incidence rates were Andorra (649.8 per 100,000), Norway (522.7 per 100,000), and Switzerland (512.6 per 100,000). For femoral fractures, the countries with the highest incidence rates were Slovenia (439.5 per 100,000), Afghanistan (396.4 per 100,000), and Croatia (375.2 per 100,000). For patella, tibia or fibula, or ankle fractures, the three countries with the highest incidence rates were Slovenia (1167.7 per 100,000), Saudi Arabia (1167.7 per 100,000), and New Zealand (1135.1 per 100,000). For foot bone fractures, the countries with the highest incidence rates were New Zealand (823.5 per 100,000), Australia (697.0 per 100,000), and Uruguay (587.0 per 100,000). Additionally, Russia exhibited high ASIRs for LEPFs, excluding hip and foot bone fractures. Countries in Asia, Middle East, North Africa, and Latin America showed notably high ASIRs for fractures of the pelvis and femur (Figure 2). In terms of the EAPC of ASIR, total LEPFs and all subtypes showed the highest average annual percentage increases in Middle Eastern and African countries, such as Syria, Libya, and Yemen. Notably, for hip fractures, marked increases were also observed in some high-income countries, including Australia, Canada, and the United States. (Supplementary Figure S4).

Regarding YLDs, in 2021, the highest numbers for total LEPFs and all subtypes were observed in China, India, and the United States (Supplementary Figure S5). The percentage change in YLDs between 1990 and 2021 showed an overall increase in most countries, with the highest increases again seen in the Middle East and Africa (Supplementary Figure S6). For ASYR, considerable variation was observed across countries, with the highest-ranking countries showing a distribution pattern similar to that of ASIR (Supplementary Figure S7). Regarding the EAPC of ASYR, most countries experienced a decline in total LEPFs and their subtypes. However, increases were noted in some Middle Eastern and African countries. For hip fractures specifically, countries in North America and Oceania—such as the United States, Canada, and Australia—showed increasing trends (Supplementary Figures S8, S14).

### Age-specific epidemiology and burden of LEPFs

**Figure 3:**
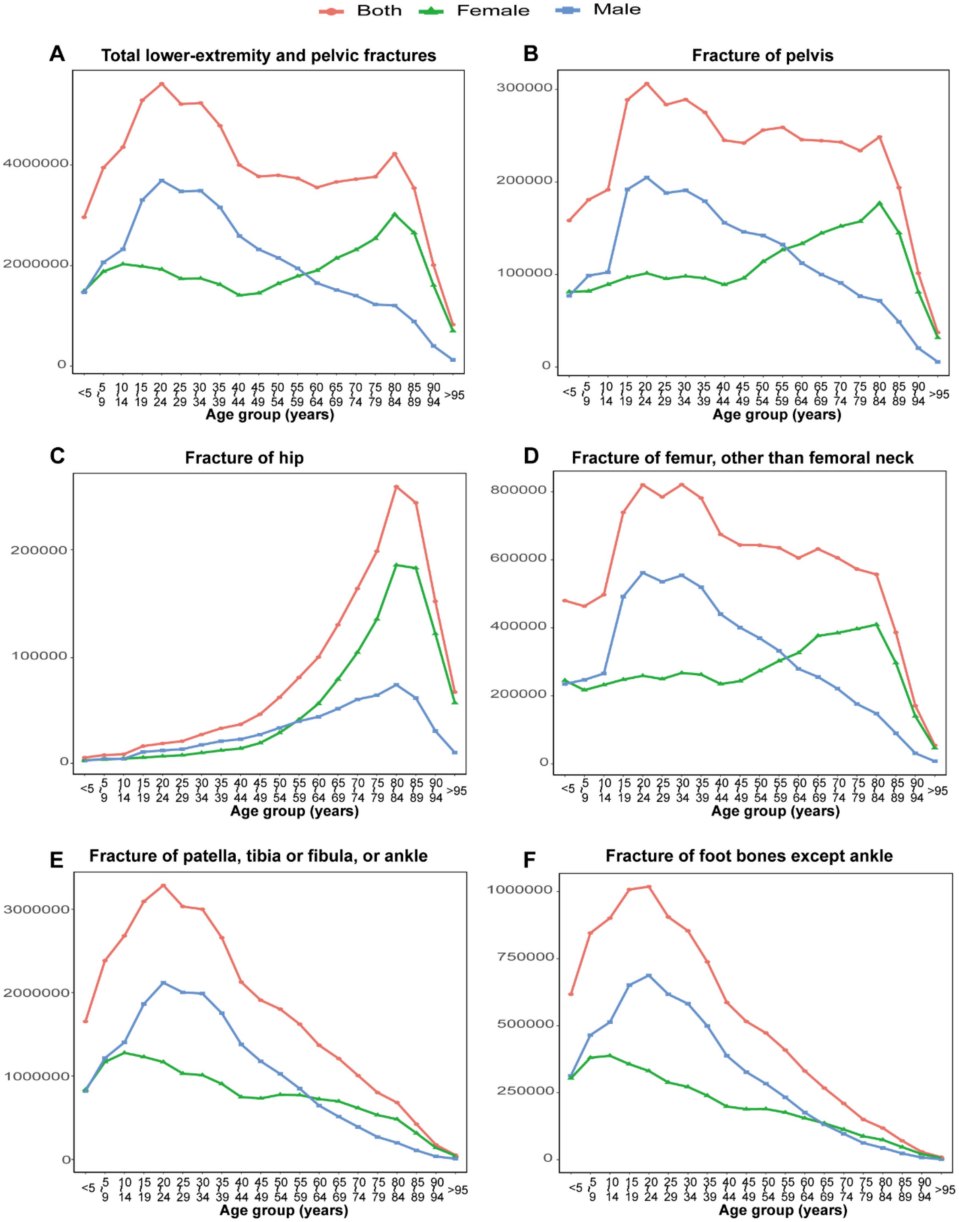
The number of incident cases for total LEPFs and anatomical subtypes by age groups in 2021. **(A)** The total LEPFs. **(B)** Fracture of pelvis. **(C)** Fracture of hip. **(D)** Fracture of femur, other than femoral neck. **(E)** Fracture of patella, tibia or fibula, or ankle. **(F)** Fracture of foot bones except ankle. LEPFs, lower extremity and pelvic fractures.

In 2021, the peak age of incident cases for LEPFs varies by fracture site and gender. The peak age of incident cases for total LEPFs, as well as pelvic and femur fractures, was 20–24 years in males and 80–84 years in females. For hip fractures, the peak age was 80–84 years in both sexes. For fractures of the patella, tibia or fibula, or ankle, as well as foot bone fractures, the peak age was 20–24 years in males and 10–14 years in females (Figure 3). In terms of ASIR, in 2021, total LEPFs and all subtypes—except for fractures of the patella, tibia or fibula, or ankle, and foot—showed higher rates in older age groups, with a sharp increase after age 60. For the two exceptions, ASIR exhibited two peaks: at ages 15–24 and 85–95 (Supplementary Figure S9).

For YLDs, the peak age group for total LEPFs and all subtypes was 45–60 years. ASYR increased progressively with age for total LEPFs and all subtypes (Supplementary Figures S10, 11).

### Primary causes of LEPFs

**Figure 4:**
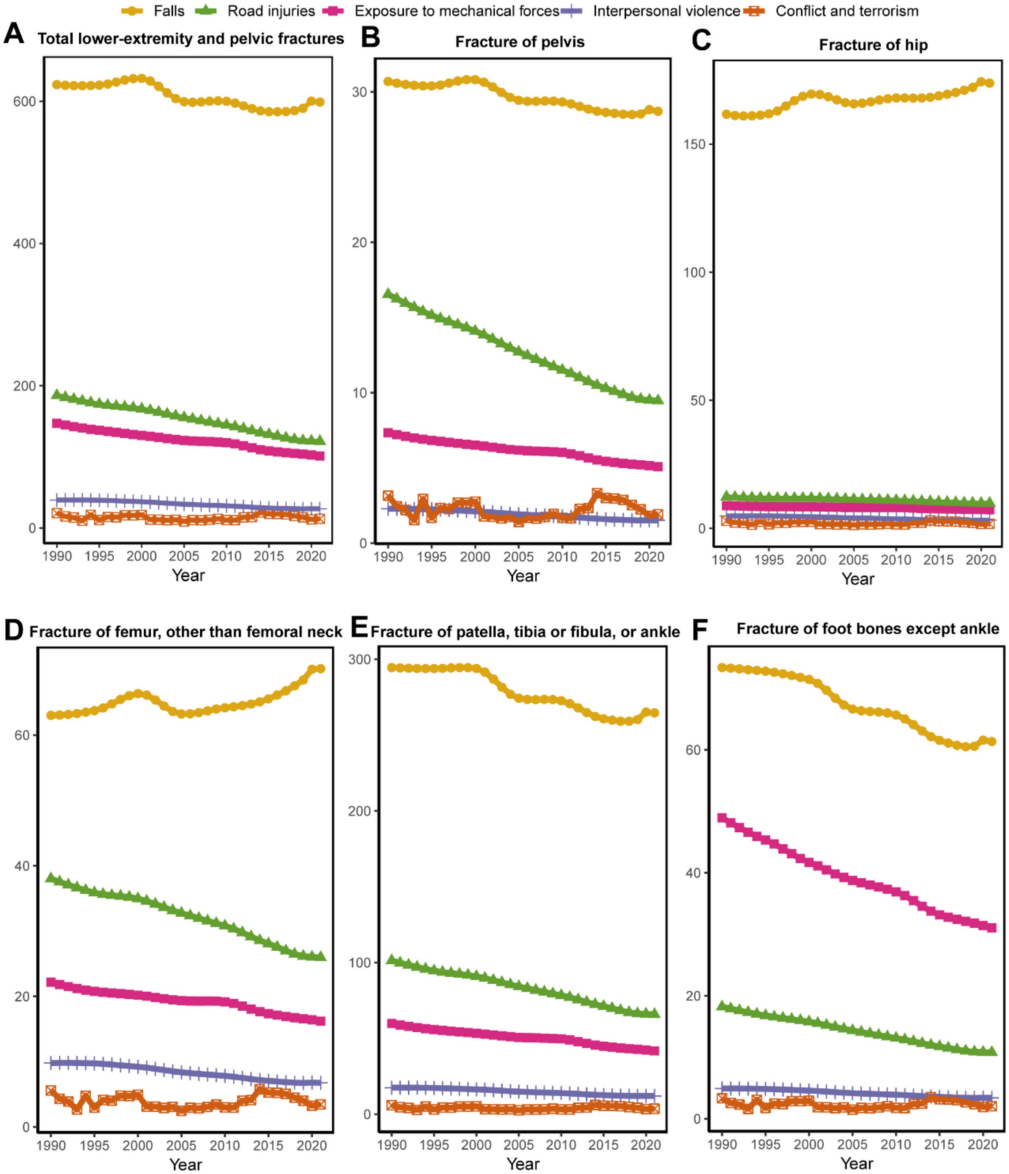
Top 5 causes of ASIR for total LEPFs and anatomical subtypes globally from 1990 to 2,121. **(A)** The total LEPFs. **(B)** Fracture of pelvis. **(C)** Fracture of hip. **(D)** Fracture of femur, other than femoral neck. **(E)** Fracture of patella, tibia or fibula, or ankle. **(F)** Fracture of foot bones except ankle. ASIR, age-standardized incidence rate; LEPFs, lower extremity and pelvic fractures.

From 1990 to 2021, for ASIR, falls were the leading cause of total LEPFs and all anatomical subtypes, followed by road injuries and exposure to mechanical forces. For total LEPFs and pelvic fractures, the ASIR attributable to falls increased from 1990 to 2000, but began to decline after 2000. For hip and femur fractures, the ASIR due to falls increased from 1990 to 2000, decreased from 2000 to 2005, and then began to rise again after 2005. For Fractures of the patella, tibia or fibula, or ankle, and foot, the ASIR attributable to falls declined from 1990 to 2019, but has increased in recent years. Overall, over the past 32 years, the ASIRs due to falls for hip and femoral fractures have exhibited an upward trend, whereas those for total LEPFs and other anatomical subtypes have shown an overall decline. Additionally, the ASIR attributable to road injuries and mechanical forces consistently declined from 1990 to 2021 for total LEPFs and all anatomical subtypes. Notably, for foot bone fractures, the ASIR attributable to exposure to mechanical forces was higher than that due to road injuries, making it the second leading cause (Figure 4).

In terms of ASYR, falls were the leading cause of total LEPFs and all anatomical subtypes. For total LEPFs and all subtypes except for fractures of the hip and femur, the ASYR attributable to falls declined from 1990 to 2019 but has shown upward trend in recent years (2019–2021). For hip fractures, the ASYR due to falls decreased from 1990 to 1995, increased from 1995 to 2000, and then continued to decline thereafter. For femoral fractures, the ASYR attributable to falls increased from 1990 to 2000, decreased from 2000 to 2005, and subsequently continued to rise—demonstrating an overall increasing trend over the past 32 years. In contrast, the ASYR attributable to road injuries and exposure to mechanical forces consistently declined from 1990 to 2021 for total LEPFs and all anatomical subtypes. (Supplementary Figure S12).

### Correlation analysis between SDI and LEPFs

**Figure 5:**
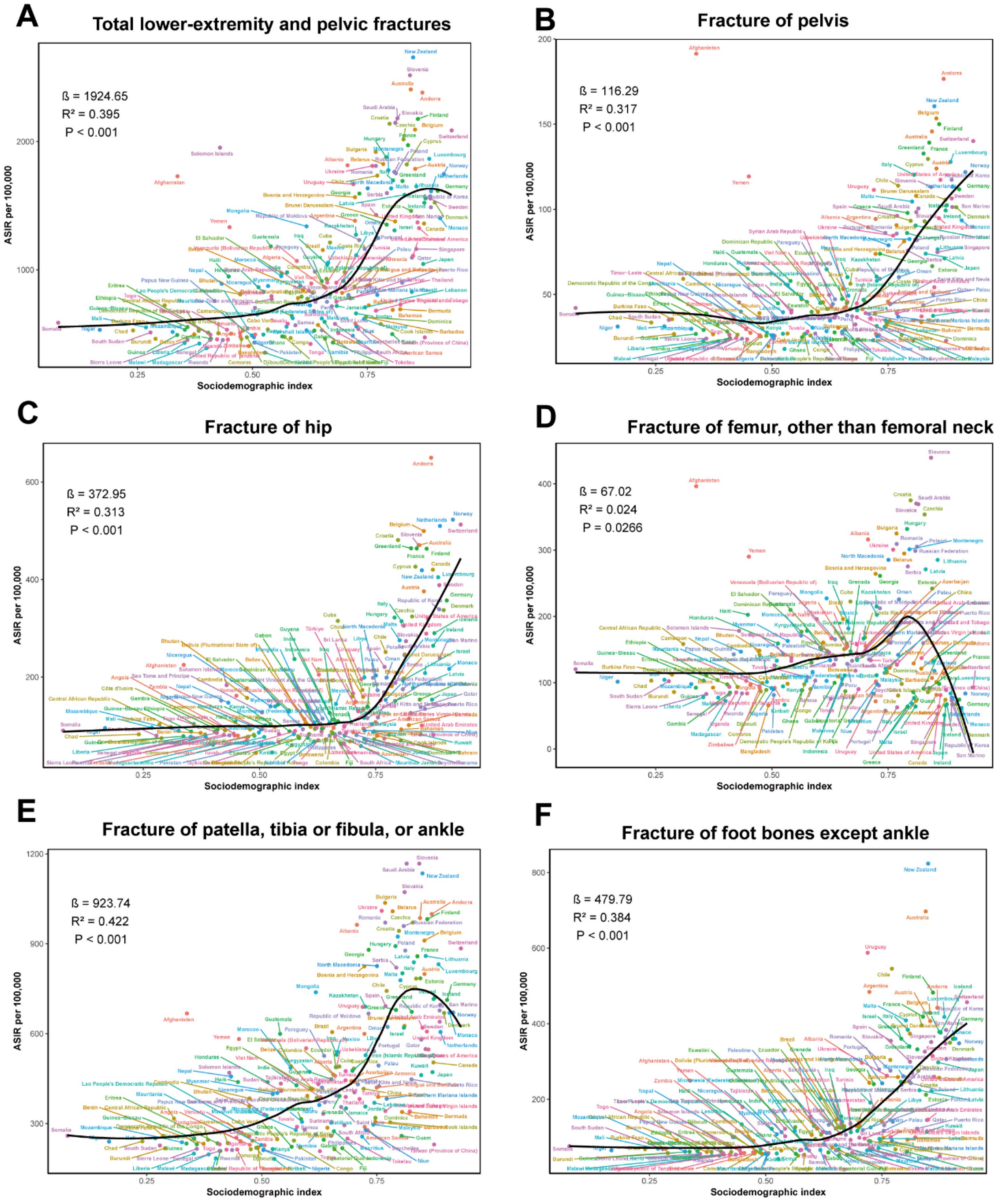
The correlation between SDI and ASIR for total LEPFs and anatomical subtypes across 204 countries and territories. **(A)** The total LEPFs. **(B)** Fracture of pelvis. **(C)** Fracture of hip. **(D)** Fracture of femur, other than femoral neck. **(E)** Fracture of patella, tibia or fibula, or ankle. **(F)** Fracture of foot bones except ankle. ASIR, age-standardized incidence rate; LEPFs, lower extremity and pelvic fractures; SDI, Socio-demographic Index.

The relationship between SDI and the epidemiology and burden of LEPFs varied across anatomical subtypes (Figure 5; Supplementary Figure S13). In terms of ASIR, total LEPFs showed a moderate positive correlation with SDI (*β* = 1964.25, *R*^2^ = 0.395, *p* < 0.001) (Figure 5A). Among anatomical subtypes, fractures of the pelvis (*β* = 116.29, *R*^2^ = 0.371, *p* < 0.001), hip (*β* = 372.95, *R*^2^ = 0.313, *p* < 0.001), and foot bones (*β* = 479.79, *R*^2^ = 0.384, *p* < 0.001) also showed an overall positive association with SDI; however, the slope remained relatively flat when SDI was below 0.7, with a sharp increase observed beyond 0.7 (Figures 5B,C). Femur fractures demonstrated a slight increase in ASIR with SDI below 0.8, followed by a notable decline beyond this threshold, resulting in an overall non-significant correlation (*β* = 67.02, *R*^2^ = 0.024, *p* = 0.0266) (Figure 5D). In contrast, fracture of patella, tibia or fibula, or ankle showed the strongest positive correlation with SDI (*β* = 923.74, *R*^2^ = 0.422, *p* < 0.001), with ASIR peaking at an SDI value of approximately 0.8 (Figure 5E).

In terms of ASYR, the association patterns with SDI were largely consistent with those observed for ASIR. However, for hip fractures, the relationship between ASYR and SDI did not reach statistical significance (*β* = 16.88, *R*^2^ = 0.03, *p* = 0.0131) (Supplementary Figure S13).

## Discussion

LEPFs represent a significant global public health issue, contributing to considerable social and economic burdens. Although common, the global epidemiology and burden of LEPFs remain insufficiently characterized. Understanding the temporal trends, geographic distribution, and primary causes of LEPFs can help policymakers implement targeted interventions and allocate resources more effectively. This study provides a comprehensive analysis of LEPFs and their anatomical subtypes at the global and national levels, covering epidemiology and disease burden by age and sex, primary causes, and correlations with SDI.

### Global epidemiology and burden of LEPFs

From 1990 to 2021, the incident cases and YLDs of LEPFs and all anatomical subtypes increased, largely driven by population growth. Over the past 32 years, the ASIRs for total LEPFs and most subtypes, as well as the ASYRs for total LEPFs and all anatomical subtypes, have declined, reflecting effective injury prevention and improved fracture care. Notably, incident hip fractures increased by 126% from 1990 to 2021, nearly triple the rate of population growth (45.7%) (13), while the ASIR remained stable. Given the well-established causal relationship between osteoporosis and hip fractures, with a 10% loss of bone mass can result in a 2.5 times greater risk of hip fracture (15), these trends suggest inadequate osteoporosis screening, prevention and treatment, and worsening bone health among elders (16, 17). For instance, in the UK, despite a growing aging population, the annual prescription rates of anti-osteoporosis medications have not shown a proportional rise since 2006 (18). With global population aging, the absolute number of hip fractures is expected to rise substantially, making the implementation of comprehensive osteoporosis prevention and treatment programs—including lifestyle intervention, exercise, universal bone density screening, vitamin D and calcium supplementation, and pharmacological intervention for high-risk individuals—an urgent public health priority (10).

### Epidemiology and burden of LEPFs across different countries

The epidemiology and burden of LEPFs vary significantly across countries, likely reflecting differences in culture, population demographics, economic development, and healthcare infrastructure. In terms of incident cases and YLDs, China, India, and the United States reported the highest numbers, likely due to their large populations. In contrast, countries such as Australia, Slovenia, and New Zealand exhibited the highest ASIR and ASYR, potentially due to higher rates of osteoporosis and participation in high-risk activities (e.g., skiing, extreme sports) (19, 20). Notably, Russia also exhibited high ASIR and ASYR, potentially associated with vitamin D deficiency from limited sunlight exposure (21, 22), harsh winter conditions that exacerbate fall risks (21), demographic aging, and the high prevalence of modifiable risk factors such as smoking and alcohol consumption (23).

Over the past three decades, countries in the Middle East and Africa have experienced a marked escalation in the burden of LEPFs, plausibly linked to sustained armed conflicts. Nations including Syria, Yemen, Libya, and Afghanistan—characterized by high war-related morbidity and mortality since 1989 (24) —showed the most pronounced increases in ASIR and ASYR. Prior studies has linked armed conflict to a heightened risk of limb fractures (25), aligning with our findings. Moreover, conflicts frequently compromise healthcare infrastructure, impeding access to timely surgical and rehabilitative care and contributing to greater disability (26). In addition, for ASIR and ASYR of hip fractures, marked increases in were also observed in some high-income countries, including Australia, Canada, and the United States. As fragility fractures are closely associated with osteoporosis, these trends reflect the growing burden of osteoporosis in aging populations in these developed countries. Reports indicate that over one-third of postmenopausal women in North America are affected by osteoporosis (27), yet treatment rates remain suboptimal, with only 25% of individuals at high fracture risk receiving appropriate anti-osteoporotic therapy (28, 29).

### Sex differences in the epidemiology and burden of LEPFs

Sex-specific disparities were evident in LEPFs burden. ASIR and ASYR for total LEPFs and most subtypes (excluding hip fractures) were consistently higher in males, likely attributed to increased exposure to occupational hazards, high-energy trauma, and risk-prone behaviors (30). Previous studies have demonstrated that young and middle-aged men are disproportionately affected by trauma-related LEPFs due to their social roles and lifestyle patterns (31, 32). Conversely, women demonstrated higher rates for hip fractures, predominantly driven by osteoporosis rather than external trauma (33). Globally, osteoporosis affects 23.1% of women, and one in three women aged >50 years will sustain an osteoporotic fracture in their lifetime (10, 34, 35). These findings underscore the critical need for implementing evidence-based preventive and therapeutic strategies in osteoporosis management for this high-risk population.

Although the ASIR and ASYR for total LEPFs and most anatomical subtypes (except hip fractures) have declined for both sexes, the rate of decline was more pronounced in males. This can be attributed to enhanced injury prevention strategies that predominantly benefit male-dominated occupations and behaviors (36). Stricter occupational safety laws, improvements in traffic regulations, and the normalization of protective sports practices have all contributed to a more rapid decline in trauma-related fractures among men (36, 37). The ASIR for hip fractures among males has shown a significant upward trend, while remaining relatively stable among females. This discrepancy may be attributed to the long-standing ignore of osteoporosis in men, who are less likely to be screened, diagnosed, or treated for osteoporosis compared to women (38, 39). A study reported that as many as 86.88% of men with osteoporosis in the US remain undiagnosed, a proportion significantly higher than that of women (64.05%) (40). Furthermore, the increasing burden of comorbidities and sarcopenia in aging men contributes to heightened fall risk, potentially exacerbating the burden of hip fractures (41). These findings underscore the urgent need for gender-inclusive osteoporosis management and fall prevention strategies (42, 43).

### Age-specific epidemiology and burden of LEPFs

In 2021, the age-specific distribution of LEPFs varied significantly by sex and anatomical site. Incidence in males peaked at ages 20–24, likely due to higher exposure to high-energy trauma from occupational, sports, and traffic-related activities (31, 44). In contrast, females exhibited a peak at ages 80–84, particularly for pelvic, hip, and femur fractures, reflecting a higher susceptibility to fragility fractures (45). ASIR for total LEPFs and hip/femur fractures rose sharply after age 60 in both sexes, highlighting the impact of aging and osteoporosis on fracture risk. PTFA and foot fractures showed bimodal peaks at 15–24 and 85–95 years, corresponding to high-energy trauma in youth and low-energy falls in the older adult.

YLDs peaked between ages 45–60 across subtypes, a period of high economic productivity, indicating substantial socioeconomic burden. ASYR increased steadily with age, suggesting greater disability in older populations. These findings support age- targeted prevention strategies, including early bone health monitoring, fall prevention for seniors, and injury mitigation in young males.

### Primary causes of LEPFs

Our findings indicate that falls were the leading cause of total LEPFs and across all anatomical subtypes from 1990 to 2021, consistent with previous studies (13). The increase in fall-related hip and femoral fractures may reflect stagnant osteoporosis treatment, especially in men, and shortcomings in fall prevention strategies (37). This underscores the critical need for integrated strategies that simultaneously address bone strength through osteoporosis management and fall risk through environmental modifications, balance and resistance training, and medication review (10, 46). In contrast, road injuries and mechanical forces declined over time, likely due to improved traffic safety and injury control measures (28). However, mechanical forces remained the second leading cause for foot bone fractures, pointing to persistent occupational or sport-related risks attached to foot bone fractures.

Of note, the overall ASYR and ASYR attributed to falls declined before 2019 but showed an upward trend in recent years, possibly linked to reduced accessibility of healthcare services for fracture during the COVID-19 pandemic. Studies have indicated that the COVID-19 pandemic and national lockdowns did not result in a reduction in fracture incidence, but rather led to a significant decrease in fracture-related hospital admissions (47, 48). For example, in Norway, elective hip surgeries fell to one-third, emergency hip surgeries in men were reduced by half, and Portugal reported an 83% decrease in fracture surgeries during lockdown (47, 48). These findings may provide important lessons for future health policy decisions and highlight the need for resilient health policies to address fracture care during public health crises. Notably, in addition to the effects of the COVID-19 pandemic on the burden of LEPFs identified in this study, its impact on the burden of other diseases, including diabetes, autoimmune disorders and Alzheimer’s disease, has also been confirmed by previous studies (49, 50).

### Correlation analysis between SDI and LEPFs

Overall, higher SDI was associated with increased ASIR and ASYR of LEPFs, with this positive correlation becoming more pronounced when SDI exceeded 0.7. This trend likely attributes to aging populations, lower bone mineral density, greater exposure to risk factors such as traffic accidents and falls, and more advanced diagnostic and reporting systems in high-SDI regions. Therefore, targeted prevention strategies are essential for high-SDI countries, particularly those with an SDI above 0.7.

## Limitation

Despite our study provided these valuable findings, it is crucial to acknowledge inherent limitations of the study. Firstly, GBD estimates are derived from existing primary data (e.g., surveys, hospital records, and literature reports) using complex modeling techniques to extrapolate disease burden across countries and regions. Although GBD 2021 has implemented numerous measures to enhance accuracy, model-based estimates often exhibit a wide range of uncertainty. Secondly, incomplete medical record systems and limited healthcare accessibility for LEPFs in less developed countries may contribute to an underestimation of their actual disease burden. Thirdly, the lag effect of GBD data should be noted.

## Conclusion

This study provides a comprehensive global analysis of the epidemiology, burden, and causes of LEPFs over the past 32 years. Although age-standardized incidence and disability rates have generally declined, the absolute number of LEPFs continues to rise, driven by population aging and growth. Significant variations were observed across regions, sexes, and age groups, with hip fractures emerging as a growing concern, especially among older men in high-SDI countries. The incidence and disability burden of LEPFs have significantly increased in conflict-affected regions, warranting attention. Falls remain the leading cause of LEPFs, underscoring the need for enhanced fall prevention and osteoporosis management. The COVID-19 pandemic further exposed vulnerabilities in fracture care systems. In summary, region-, sex- and age-specific prevention strategies tailored to the evolving trends, along with improved healthcare access for osteoporosis management, and enhanced public education specifically focused on osteoporosis awareness and the fracture-osteoporosis connection, are critical to mitigating the global burden of LEPFs.

## Data Availability

The original contributions presented in the study are included in the article/Supplementary material, further inquiries can be directed to the corresponding author/s.
