# Supplementary material for: Global epidemiology, burden, and causes of lower extremity and pelvic fractures in the past 32 years
Source: Front Public Health. 2025 Jul 30;13:1627867. doi: 10.3389/fpubh.2025.1627867 (PMC12343497; doi:10.3389/fpubh.2025.1627867)

**Supplementary Table**

**Table S1: Global YLDs and ASYR of LEPFs between 1990 and 2021, and their temporal trends.**

|  | **Number of YLDs** | | |  | **ASYR** | | |
| --- | --- | --- | --- | --- | --- | --- | --- |
|  | **1990**  **(95% UI)** | **2021**  **(95% UI)** | **PC** |  | **1990**  **(95% UI)** | **2021**  **(95% UI)** | **EAPC**  **(95% CI)** |
| **Total lower-extremity fractures** | |  |  |  |  |  |  |
| Both | 16172300 (10678441, 22988933) | 23512042 (15652451, 33318935) | 0.45 |  | 364.51 (241.5, 516.2) | 280.01 (186.46, 396.52) | -1.02 (-1.1, -0.94) |
| Female | 7332013 (4850251, 10364971) | 11296837 (7475603, 15921570) | 0.54 |  | 322.36 (213.77, 454.06) | 256.05 (169.2, 360.93) | -0.88 (-0.95, -0.8) |
| Male | 8840288 (5844410, 12602959) | 12215206 (8114217, 17372322) | 0.38 |  | 400.65 (265.58, 569.6) | 300.26 (199.58, 426.65) | -1.13 (-1.21, -1.05) |
| **Fracture of pelvis** |  |  |  |  |  |  |  |
| Both | 1752618 (1213438, 2342291) | 2241606 (1559349, 2965288) | 0.28 |  | 37.92 (26.13, 50.47) | 26.74 (18.59, 35.36) | -1.3 (-1.37, -1.23) |
| Female | 741412 (508622, 991504) | 977098 (681957, 1309000) | 0.32 |  | 31.52 (21.6, 42.03) | 22.59 (15.81, 30.35) | -1.24 (-1.31, -1.17) |
| Male | 1011207 (697973, 1359760) | 1264509 (883712, 1672883) | 0.25 |  | 44.25 (30.39, 59.2) | 30.85 (21.56, 40.77) | -1.34 (-1.42, -1.27) |
| **Fracture of hip** |  |  |  |  |  |  |  |
| Both | 1770882 (1250541, 2346921) | 3101921 (2176548, 4193623) | 0.75 |  | 46.91 (33.29, 62.39) | 37.65 (26.41, 50.88) | -0.77 (-0.82, -0.72) |
| Female | 1092807 (774097, 1465623) | 2027365 (1415075, 2744712) | 0.86 |  | 53.36 (37.73, 70.98) | 43.79 (30.55, 59.4) | -0.64 (-0.68, -0.59) |
| Male | 678075 (477249, 897703) | 1074556 (760323, 1437404) | 0.58 |  | 35.17 (24.83, 46.42) | 28.44 (19.95, 38.38) | -0.85 (-0.91, -0.8) |
| **Fracture of femur, other than femoral neck** | | |  |  |  |  |  |
| Both | 754039 (494852, 1083057) | 1181398 (779928, 1699881) | 0.57 |  | 17.28 (11.38, 25.01) | 14.14 (9.34, 20.38) | -0.77 (-0.85, -0.69) |
| Female | 357056 (233689, 521765) | 605869 (395635, 880643) | 0.70 |  | 15.94 (10.45, 23.25) | 13.56 (8.86, 19.72) | -0.6 (-0.68, -0.52) |
| Male | 396984 (260107, 562643) | 575529 (382547, 820117) | 0.45 |  | 18.1 (11.91, 26.15) | 14.32 (9.51, 20.36) | -0.93 (-1.01, -0.85) |
| **Fracture of patella, tibia or fibula, or ankle** | | |  |  |  |  |  |
| Both | 11592216 (7539516, 16739747) | 16577060 (10892513, 23822303) | 0.43 |  | 255.77 (166.75, 367.91) | 196.61 (129.22, 282.32) | -1.04 (-1.13, -0.95) |
| Female | 5029808 (3268540, 7211990) | 7529451 (4889459, 10742267) | 0.50 |  | 216.78 (141.19, 310.36) | 172.5 (111.84, 245.81) | -0.9 (-0.99, -0.82) |
| Male | 6562407 (4294856, 9479459) | 9047609 (5935964, 13045983) | 0.38 |  | 294.55 (193.33, 424.28) | 220.47 (144.85, 317.48) | -1.14 (-1.23, -1.05) |
| **Fracture of foot bones except ankle** | |  |  |  |  |  |  |
| Both | 302545 (180094, 476917) | 410057 (244113, 637840) | 0.36 |  | 6.63 (3.95, 10.42) | 4.87 (2.9, 7.58) | -1.19 (-1.27, -1.11) |
| Female | 110930 (65303, 174089) | 157054 (93477, 244948) | 0.42 |  | 4.76 (2.8, 7.44) | 3.61 (2.14, 5.65) | -1.06 (-1.13, -0.98) |
| Male | 191615 (114225, 303394) | 253003 (151671, 395935) | 0.32 |  | 8.58 (5.12, 13.55) | 6.18 (3.71, 9.66) | -1.27 (-1.36, -1.18) |

ASYR, age-standardized YLD rate; CI, confidence interval; EAPC, estimated annual percentage change; LEPFs, lower extremity and pelvic fractures; PC, percentage change; UI, uncertainty intervals; YLD, years lived with disability.

**Supplementary Figures**

**Abbreviations:** ASIR, age-standardized incidence rate; ASYR, age-standardized YLD rate; CI, confidence interval; EAPC, estimated annual percentage change; LEPFs, lower extremity and pelvic fractures; PC, percentage change; UI, uncertainty intervals; SDI, Socio-demographic Index; YLD, years lived with disability.

**Figure S1. The number of YLDs and ASYR for total LEPFs and anatomical subtypes by sex, 1990–2021 (A). The number of YLDs. (B) ASYR per 100,000 population.**


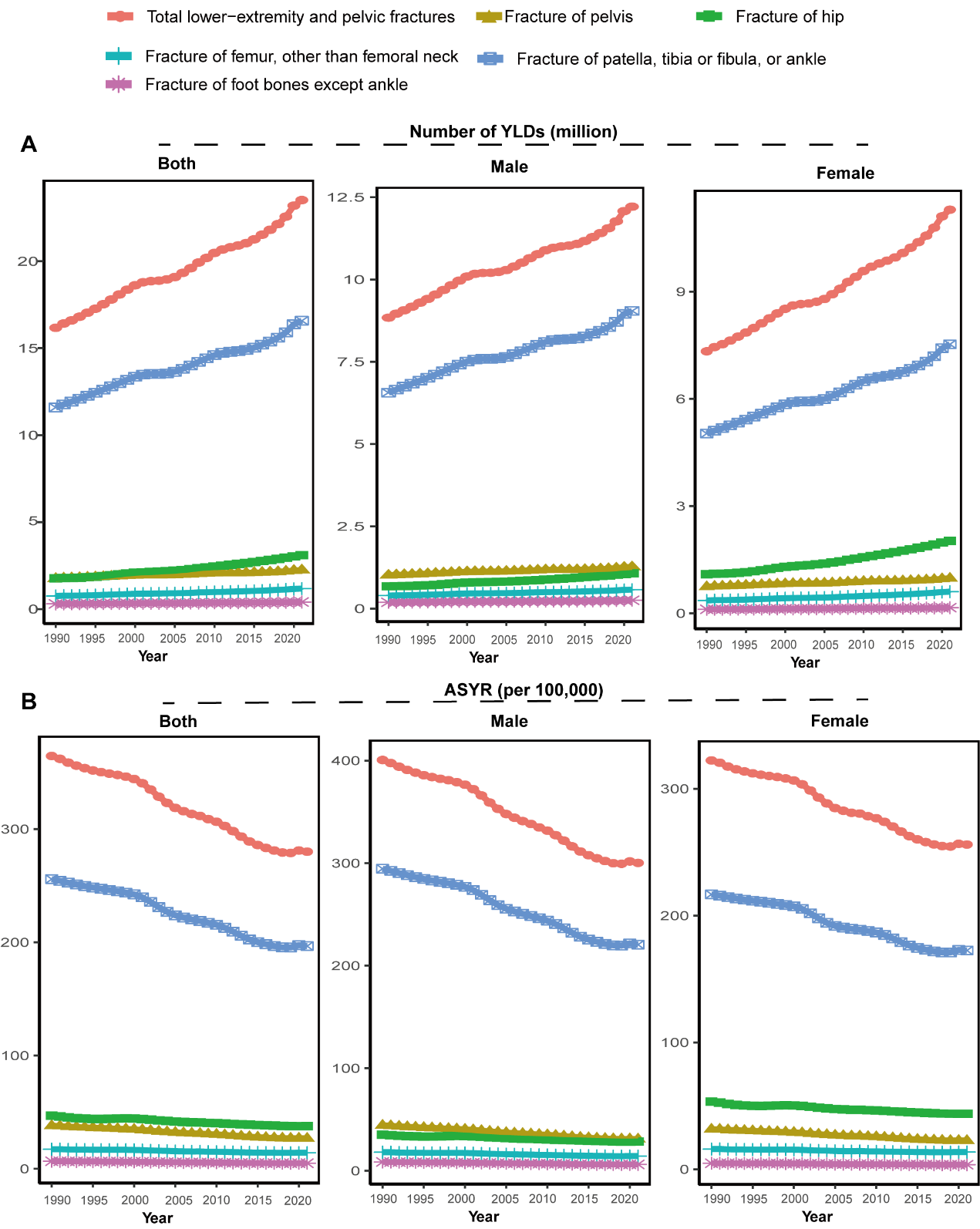


**Figure S2. The number of incident cases for total LEPFs and anatomical subtypes across 204 countries and territories in 2021. (A). The total LEPFs. (B). Fracture of pelvis. (C). Fracture of hip. (D). Fracture of femur, other than femoral neck. (E). Fracture of patella, tibia or fibula, or ankle. (F). Fracture of foot bones except ankle.**

**
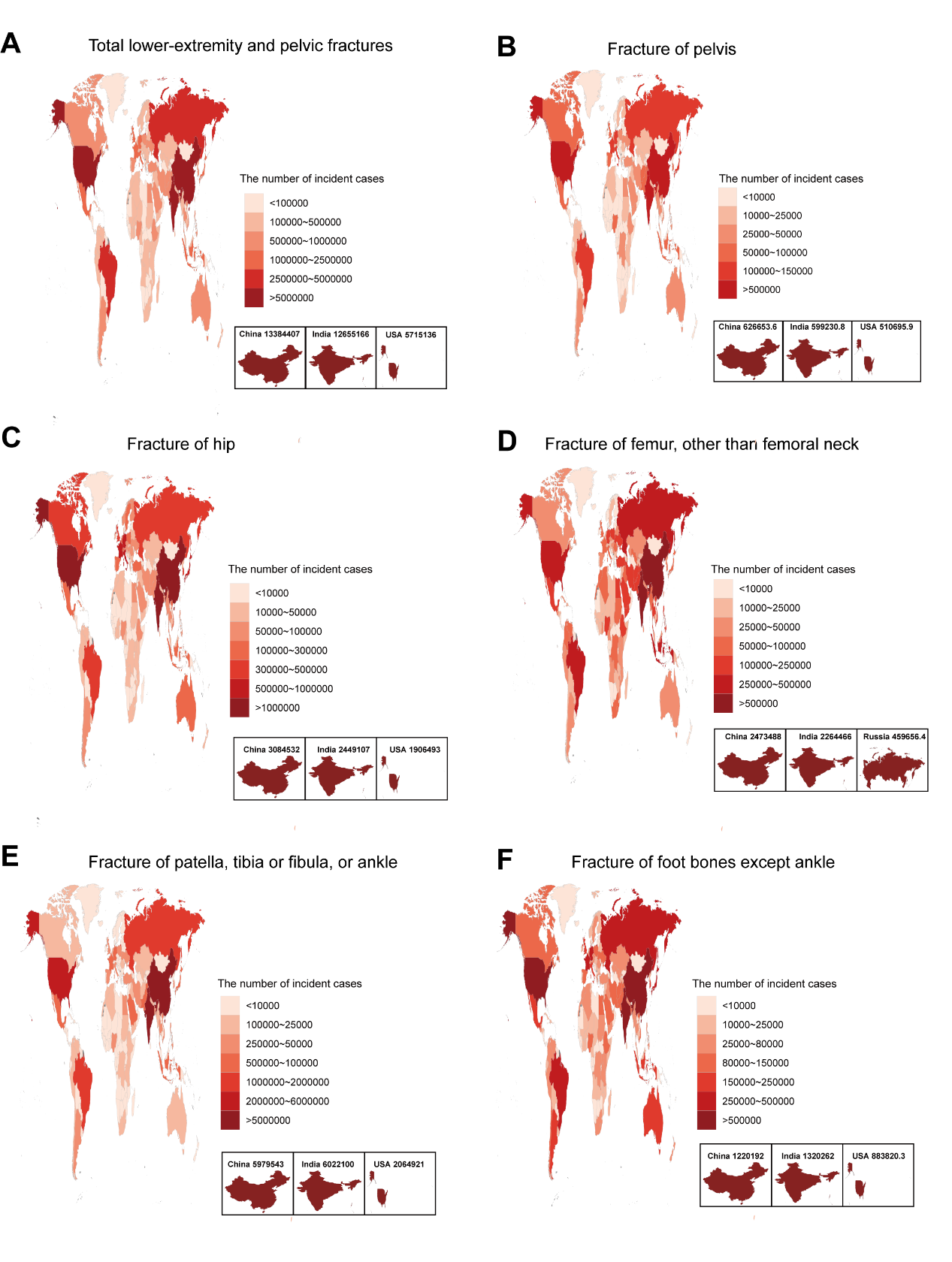
**

**Figure S3. Percentage change of the number of incident cases for total LEPFs and anatomical subtypes across** **204 countries and territories between 1990 and 2021. (A). The total LEPFs. (B). Fracture of pelvis. (C). Fracture of hip. (D). Fracture of femur, other than femoral neck. (E). Fracture of patella, tibia or fibula, or ankle. (F). Fracture of foot bones except ankle.**

**
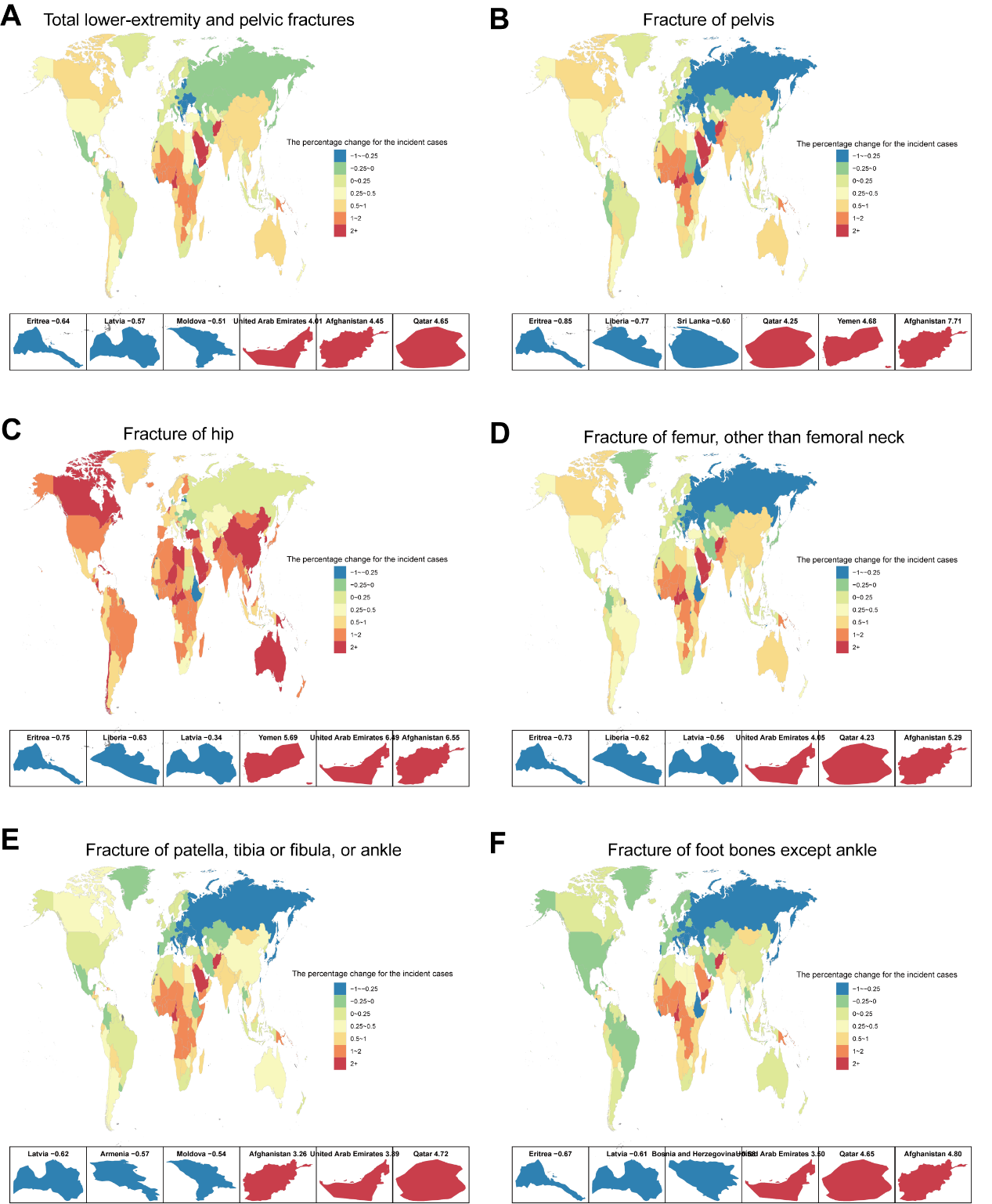
**

**Figure S4. EAPC of ASIR for total LEPFs and anatomical subtypes across 204 countries and territories over the past 32 years. (A). The total LEPFs. (B). Fracture of pelvis. (C). Fracture of hip. (D). Fracture of femur, other than femoral neck. (E). Fracture of patella, tibia or fibula, or ankle. (F). Fracture of foot bones except ankle. ASIR, age-standardized incidence rate; EAPC, estimated annual percentage change; LEPFs, lower extremity and pelvic fractures.**


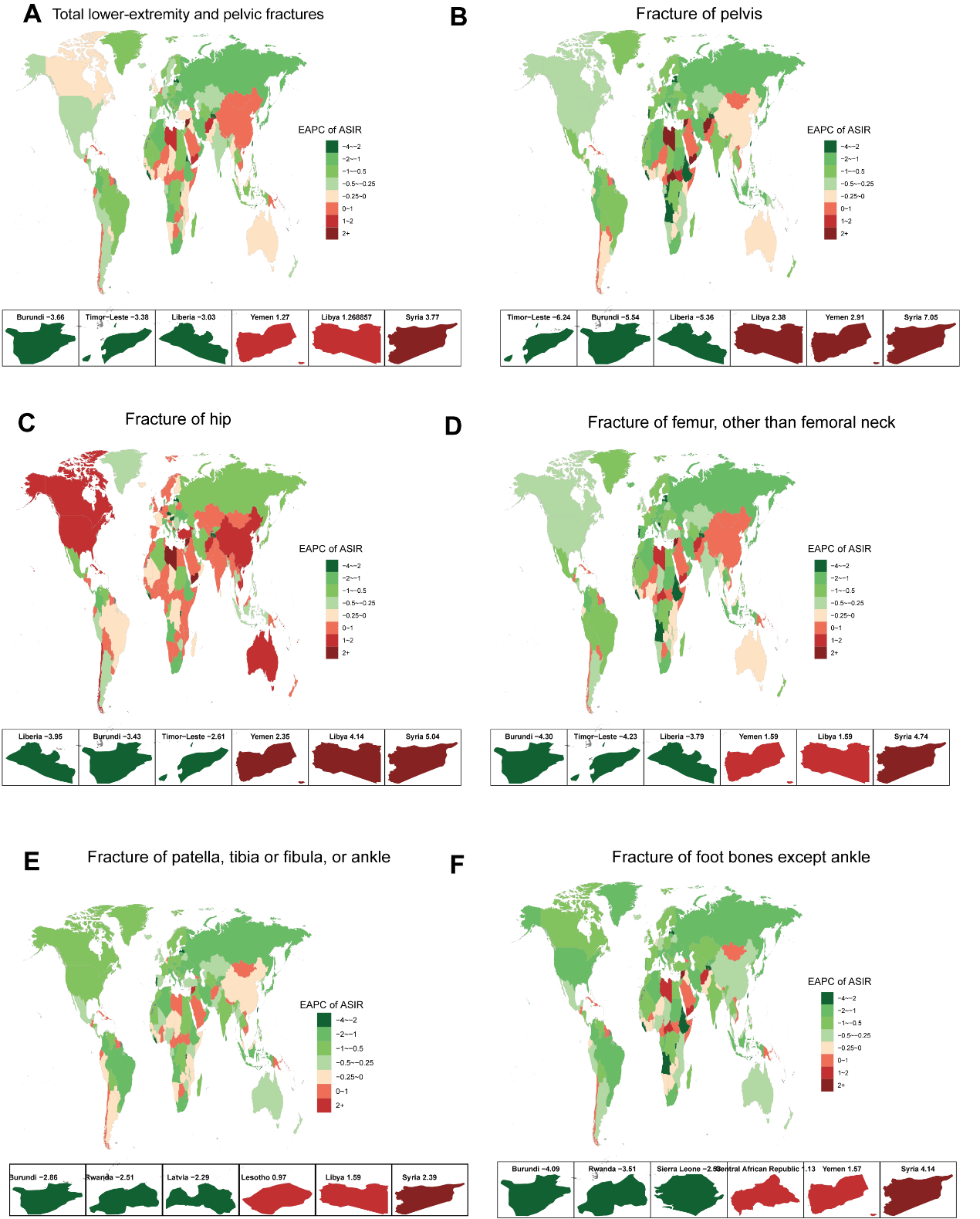


**Figure S5. The number of YLDs for total LEPFs and anatomical subtypes across 204 countries and territories in 2021. (A). The total LEPFs. (B). Fracture of pelvis. (C). Fracture of hip. (D). Fracture of femur, other than femoral neck. (E). Fracture of patella, tibia or fibula, or ankle. (F). Fracture of foot bones except ankle.**

**
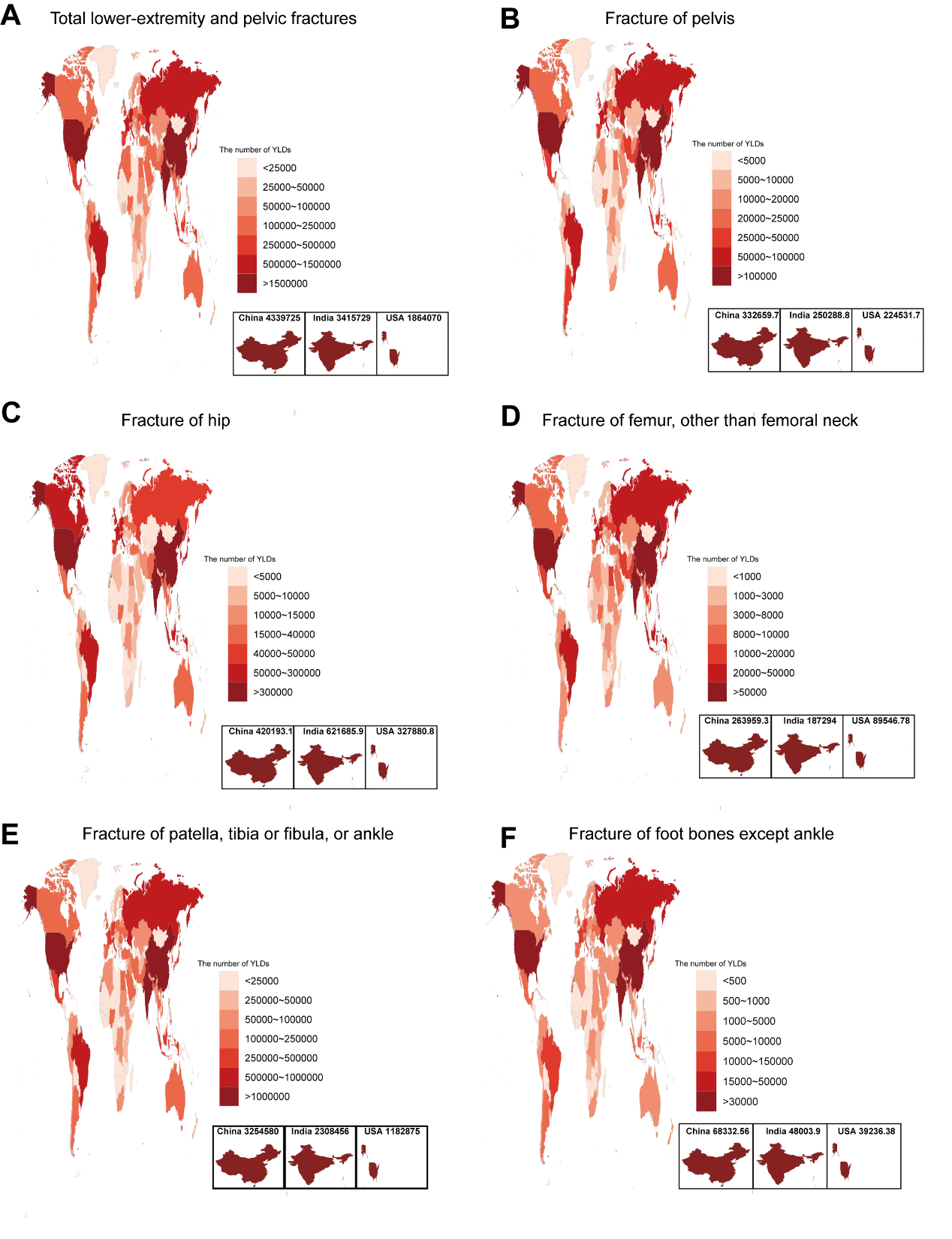
**

**Figure S6. Percentage change of the number of YLDs for total LEPFs and anatomical subtypes across 204 countries and territories between 1990 and 2021. (A). The total LEPFs. (B). Fracture of pelvis. (C). Fracture of hip. (D). Fracture of femur, other than femoral neck. (E). Fracture of patella, tibia or fibula, or ankle. (F). Fracture of foot bones except ankle.**

**
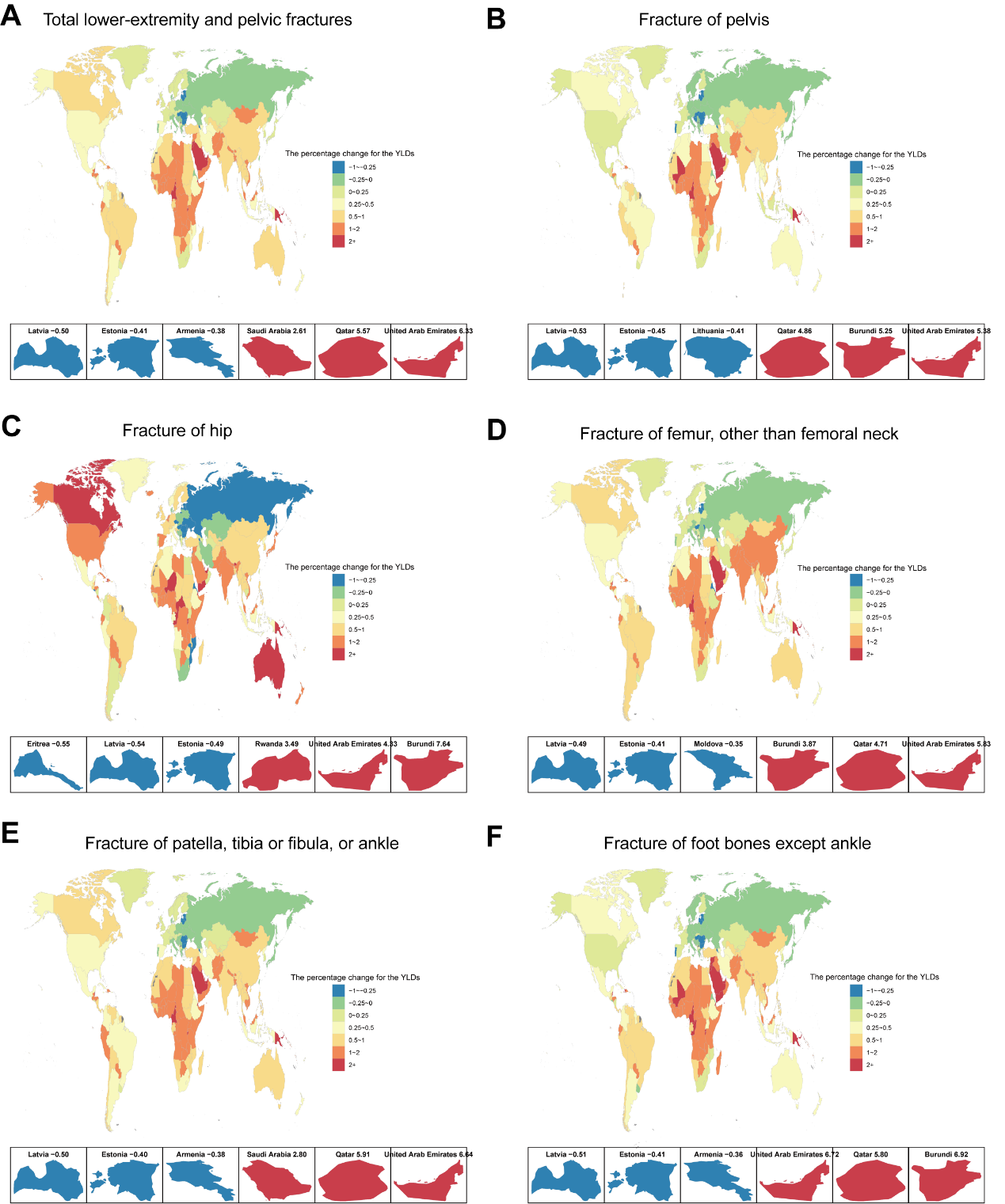
**

**Figure S7. ASYR for total LEPFs and anatomical subtypes across 204 countries and territories in 2021. (A). The total LEPFs. (B). Fracture of pelvis. (C). Fracture of hip. (D). Fracture of femur, other than femoral neck. (E). Fracture of patella, tibia or fibula, or ankle. (F). Fracture of foot bones except ankle.**

**
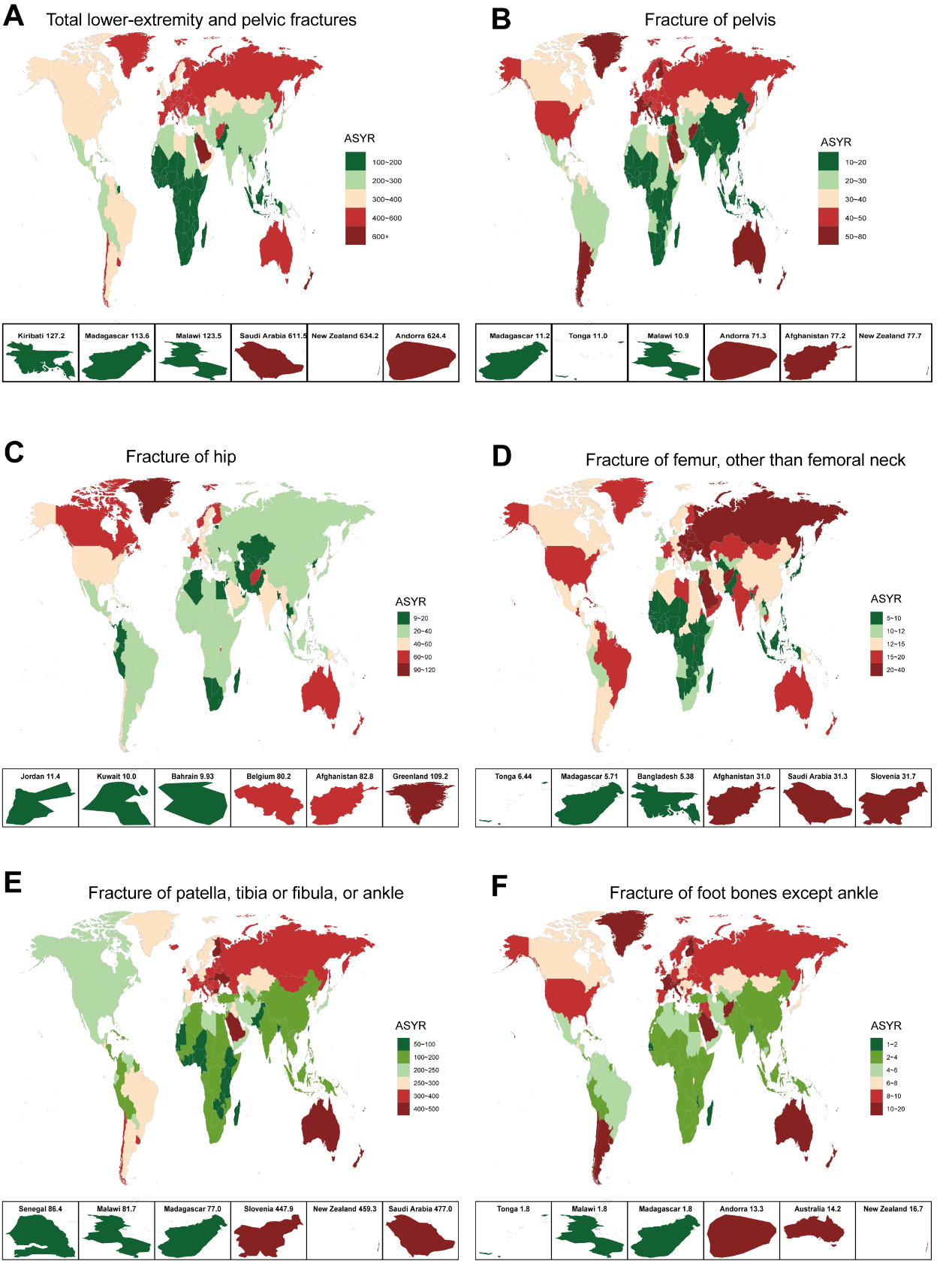
**

**Figure S8. EAPC of ASYR for total LEPFs and anatomical subtypes across 204 countries and territories in 2021. (A). The total LEPFs. (B). Fracture of pelvis. (C). Fracture of hip. (D). Fracture of femur, other than femoral neck. (E). Fracture of patella, tibia or fibula, or ankle. (F). Fracture of foot bones except ankle.**

**
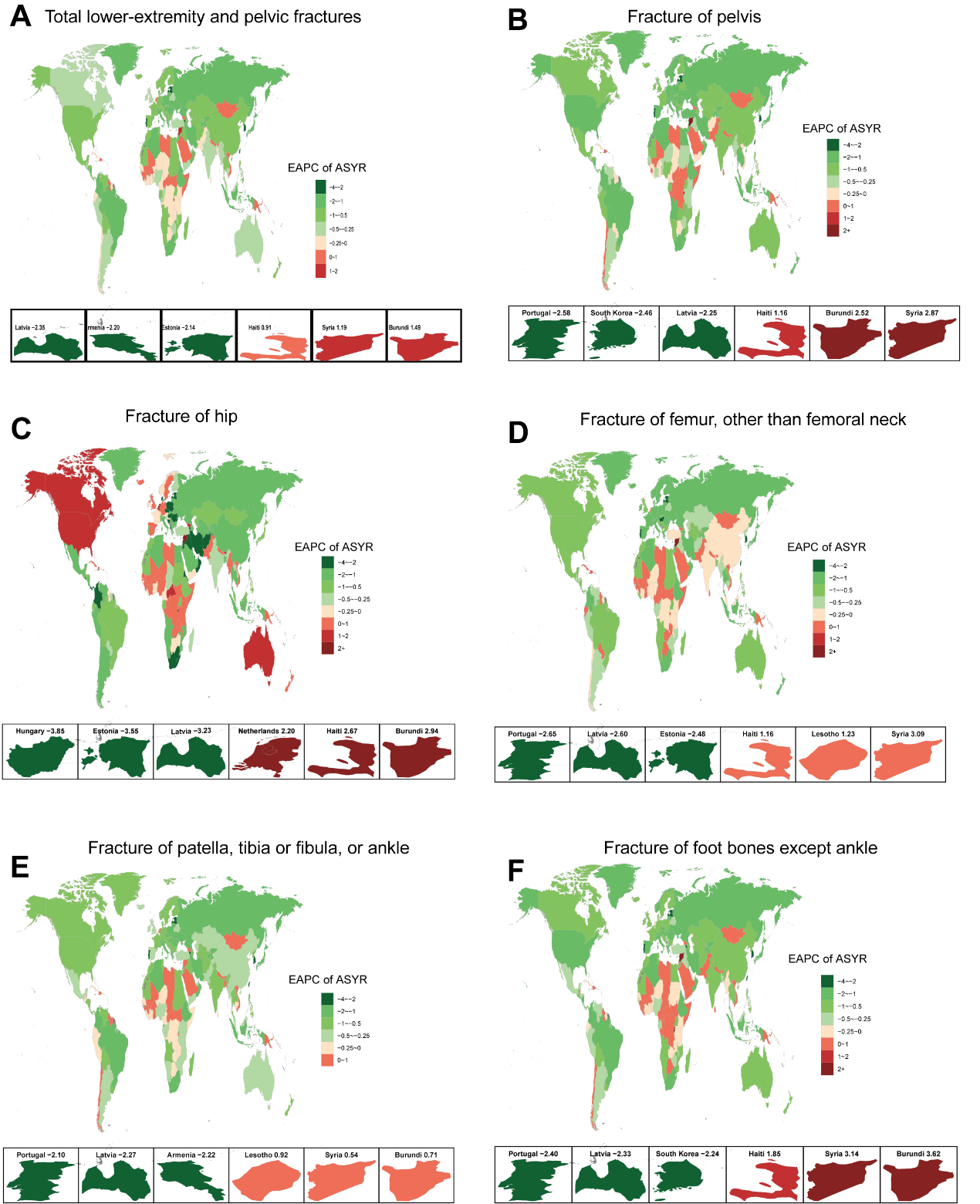
**

**Figure S9. ASIR for total LEPFs and anatomical subtypes by age groups in 2021.**

**(A). The total LEPFs. (B). Fracture of pelvis. (C). Fracture of hip. (D). Fracture of femur, other than femoral neck. (E). Fracture of patella, tibia or fibula, or ankle. (F). Fracture of foot bones except ankle.**

**
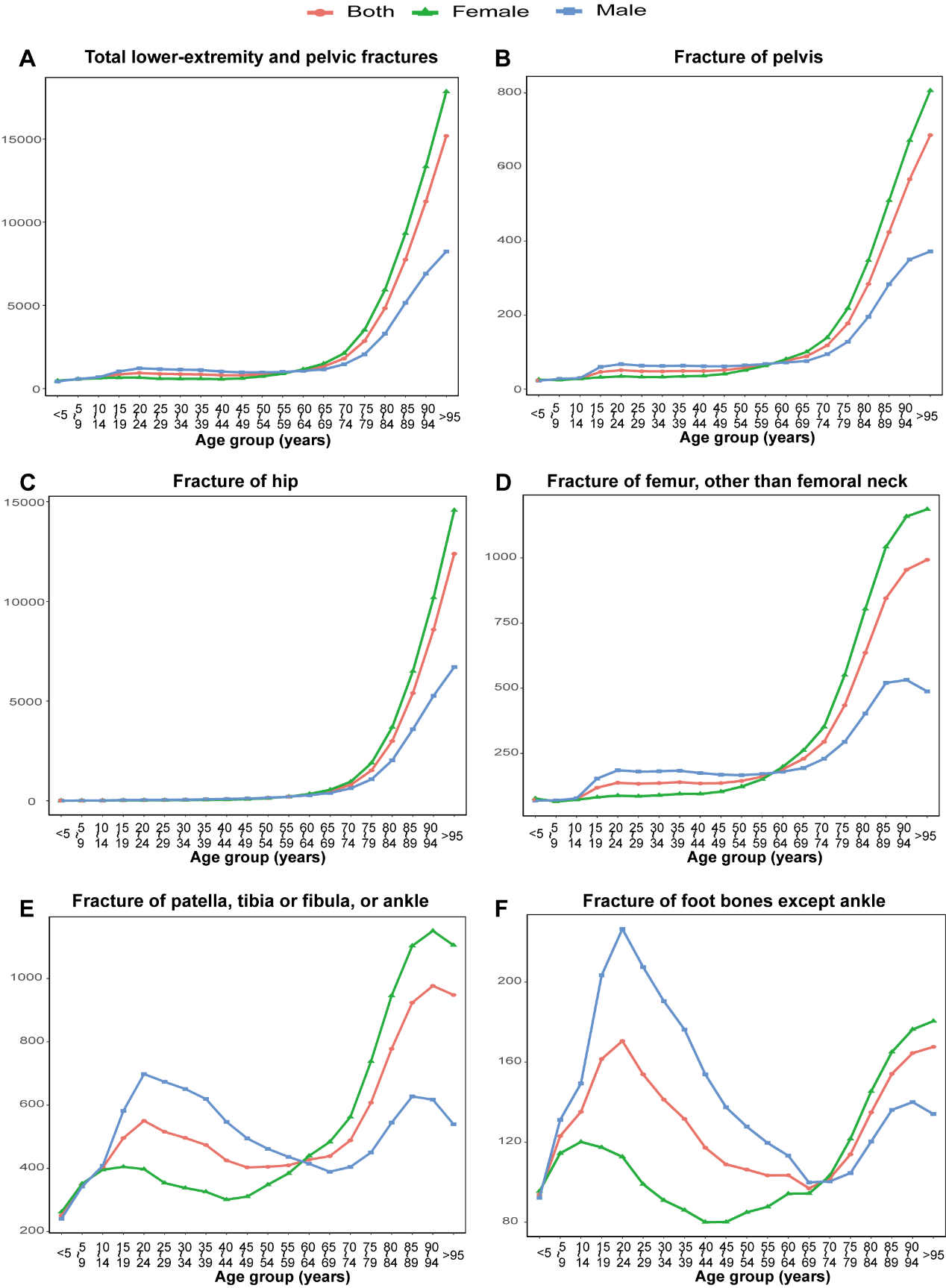
**

**Figure S10. YLDs for total LEPFs and anatomical subtypes by age groups in 2021.**

**(A). The total LEPFs. (B). Fracture of pelvis. (C). Fracture of hip. (D). Fracture of femur, other than femoral neck. (E). Fracture of patella, tibia or fibula, or ankle. (F). Fracture of foot bones except ankle.**

**
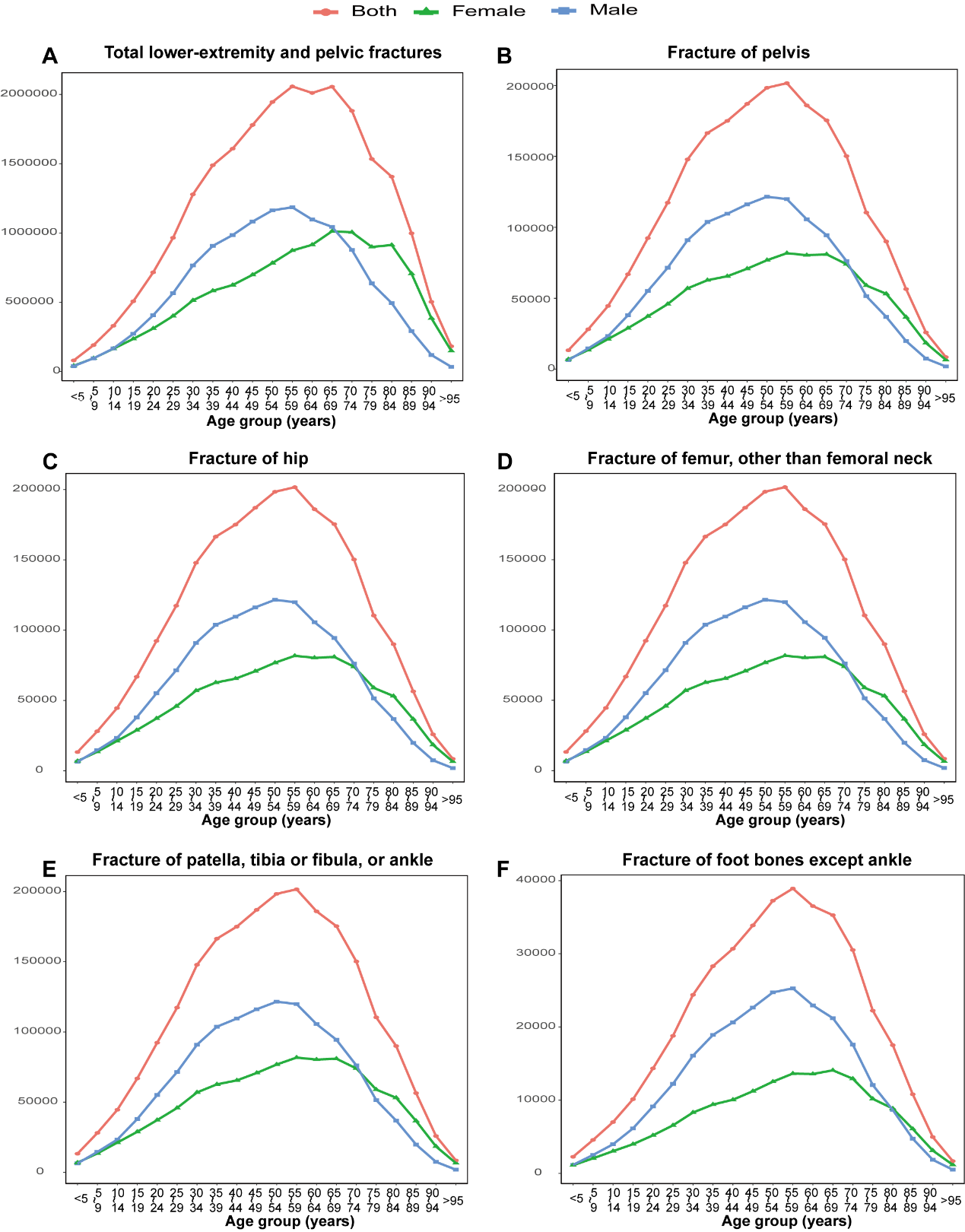
**

**Figure S11. ASYR for total LEPFs and anatomical subtypes by age groups in 2021.**

**(A). The total LEPFs. (B). Fracture of pelvis. (C). Fracture of hip. (D). Fracture of femur, other than femoral neck. (E). Fracture of patella, tibia or fibula, or ankle. (F). Fracture of foot bones except ankle.**

**
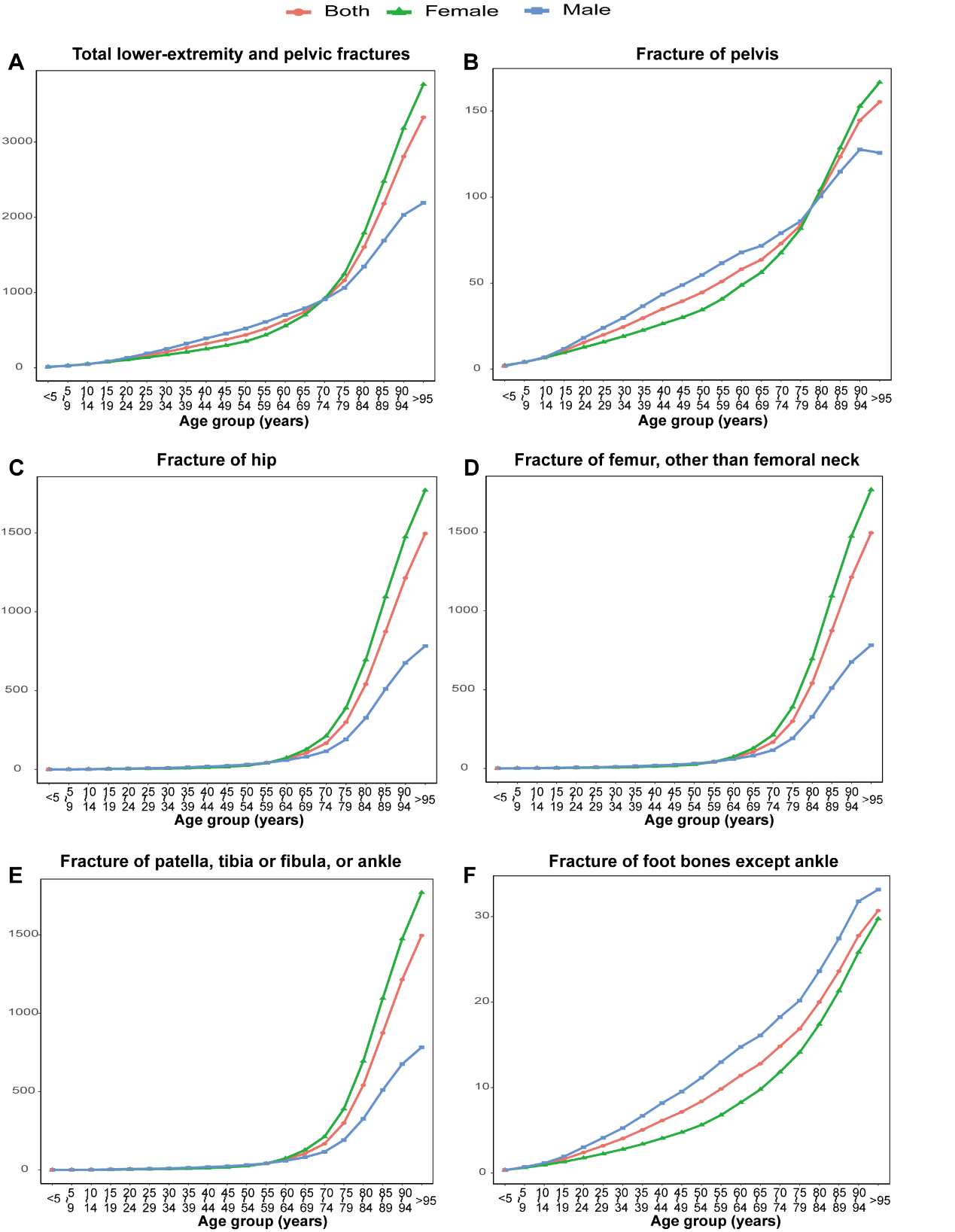
**

**Figure S12. Top 5 causes of ASYR for total LEPFs and anatomical subtypes globally from 1990 to 2121. (A). The total LEPFs. (B). Fracture of pelvis. (C). Fracture of hip. (D). Fracture of femur, other than femoral neck. (E). Fracture of patella, tibia or fibula, or ankle. (F). Fracture of foot bones except ankle.**

**
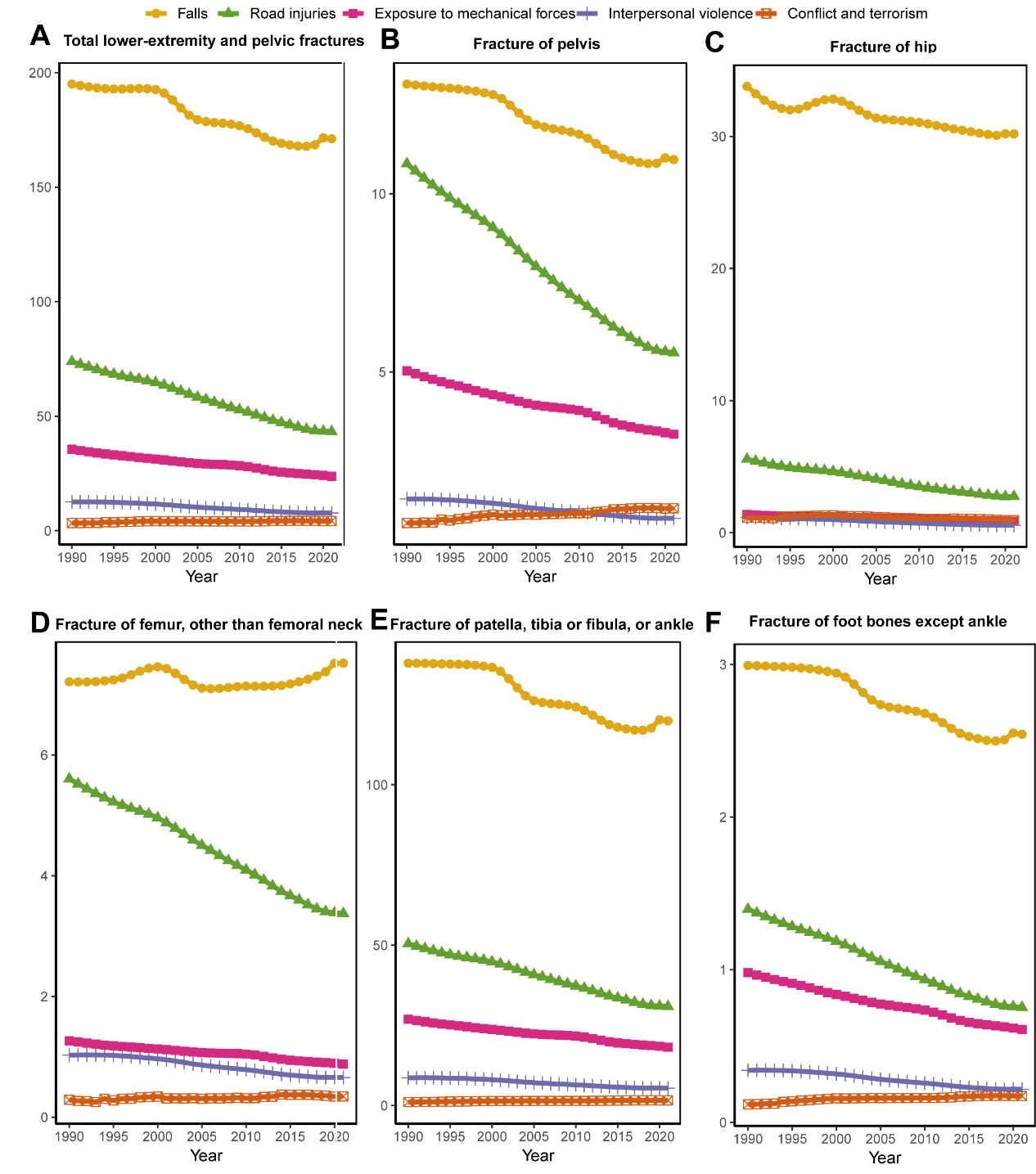
**

**Figure S13. The correlation between SDI and ASYR for total LEPFs and anatomical subtypes across 204 countries and territories. (A). The total LEPFs. (B). Fracture of pelvis. (C). Fracture of hip. (D). Fracture of femur, other than femoral neck. (E). Fracture of patella, tibia or fibula, or ankle. (F). Fracture of foot bones except ankle.**


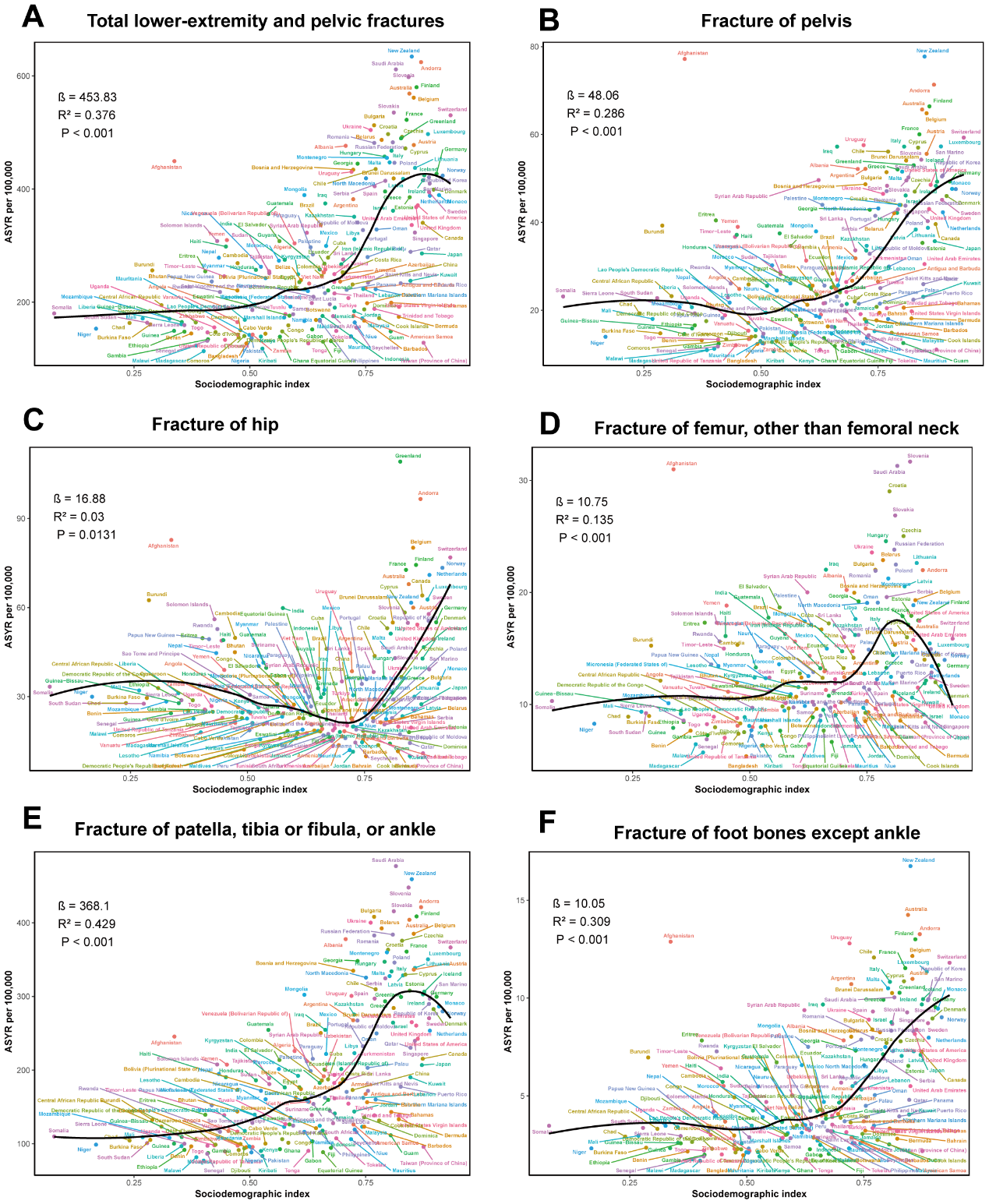

Supplement: Supplementary file 1 [file Data_Sheet_1.docx]
